# Two novel species of genus *Streptomyces* isolated from eucalyptus tissues grown in saline soil and their potential as plant growth promoters

**DOI:** 10.3389/fmicb.2026.1819523

**Published:** 2026-05-29

**Authors:** Kawinthip Kiakhunthod, Chanwit Suriyachadkun, Sumalee Chookhampaeng, Kewalee Prompiputtanaporn, Piriya Klankeo, Weerachai Saijuntha, Onuma Kaewkla

**Affiliations:** 1 Department of Biology, Faculty of Science, Mahasarakham University, Maha Sarakham Province, Thailand; 2 Thailand Bioresource Research Center (TBRC), National Center for Genetic Engineering and Biotechnology, National Science and Technology Development Agency, Klong Luang, Pathum Thani, Thailand; 3 Microscopy Section, Laboratory Service Unit (LSU), Suranaree University of Technology, Nakhon Ratchasima Province, Thailand; 4 Omics Science and Bioinformatics Center, Faculty of Science, Chulalongkorn University, Bangkok, Thailand; 5 Center of Excellence in Biodiversity Research, Mahasarakham University, Maha Sarakham Province, Thailand

**Keywords:** *Eucalyptus camaldulensis*, fungal inhibition, saline stress, *Streptomyces kalasinensis*, *Streptomyces phytorum*

## Abstract

Two endophytic actinobacteria of the genus *Streptomyces*—EKL1.1^T^ and EKS8.28^T^—were isolated from the surface-sterilized leaf and twig of a red gum tree (*Eucalyptus camaldulensis* Dehn.) grown in highly saline soil, as reported in a previous study. These strains were aerobic and feature well-developed substrate mycelia and aerial mycelia with spiral spore chains. Strains EKL1.1^T^ and EKS8.28^T^ shared the highest 16S rRNA gene sequence similarity with *Streptomyces mexicanus* NRRL B-24196^T^ (99.2%) and *Streptomyces glomeratus* LMG 19903^T^ (99.0%), respectively. The comparative genome study of strain EKL1.1^T^ and its closest type strain, *S. mexicanus* NRRL B-24196^T^, showed the highest dDDH, ANIb, and ANIm values of 31.8, 85.3, and 88.5%, respectively. The comparative genome study of strain EKS8.28^T^ and its closest type strain, *Streptomyces cynarae* HUAS 13-4^T^, had the highest dDDH, ANIb, and ANIm values of 41.8, 89.6, and 91.4%, respectively. The genotypic and phenotypic properties of strains EKL1.1^T^ and EKS8.28^T^ distinguished these two strains from the closely related species with validly published names. The name proposed for the new species of strain EKL1.1^T^ is *Streptomyces kalasinensis* (= NRRL B-65753^T^ = TBRC 19936^T^). The name proposed for the novel species of strain EKS8.28^T^ is *Streptomyces phytorum* (= NRRL B-65754^T^ = TBRC 19937^T^). Strain EKL1.1^T^ could only inhibit one fungus, *Cladosporium* sp. LB1, moderately (35%). Strain EKS8.28^T^ could inhibit *Fusarium* sp. RE1 (71.1%), *Curvularia* sp. LB12 (62.2%), and *Cladosporium* sp. LB1 (50%). Strains EKL1.1^T^ and EKS8.28^T^ could produce indole acetic acid (IAA) and solubilize phosphate. The optimum spore inoculum of strains EKL1.1^T^ and EKS8.28^T^ to promote seedling growth parameters of eucalyptus was 10^7^ and 10^8^ spores/mL, respectively. Strains EKL1.1^T^ and EKS8.28^T^ facilitated the development of eucalyptus seedlings under saline conditions. They also enhanced the growth of eucalyptus seedlings *in planta* by augmenting shoot length and fresh weight. Genome mining of these strains reveals their *in vitro* and *in planta* characteristics, including biosynthetic gene clusters of bioactive compounds and several genes associated with plant growth enhancement under stressful conditions. They can be formulated as inoculants to improve eucalyptus plantations for sustainable agriculture in the future.

## Introduction

The genus *Streptomyces* is classified within the phylum *Actinomycetota*, family *Streptomycetaceae*, as established by Waksman and Henrici (1943). This genus has more than 800 species with validly published names (accessed February 20, 2026) (Parte et al., 2020). They were predominantly found in soil and other sources, including freshwater, marine environments, deserts, and plant tissues. They are thoroughly documented as antibiotic producers and plant growth promoters. Endophytic actinobacteria are a group of actinobacteria living inside plant tissue without any harm. Recent reports have identified advantageous strains of endophytic actinobacteria in the genus *Streptomyces* and examined their antibacterial efficacy and plant growth-promoting (PGP) attributes (Quinn et al., 2020; Kaewkla et al., 2022; Kaewkla et al., 2025a). The novel species, *Streptomyces naphthomycinicus* TML10^T^, isolated from medicinal plant tissues, produces naphthomycin to inhibit methicillin-resistant *Staphylococcus aureus* (Kaewkla et al., 2024). *Streptomyces panacea* sp. nov. produces antibiotics to inhibit multidrug-resistant (MDR) clinical isolates of *Staphylococcus aureus* (Rai et al., 2025). *Streptomyces* N2A, an endophyte isolated from a soybean plant, showed *in vitro* PGP traits and inhibited a broad spectrum of phytopathogenic fungi. In addition, this strain enhanced soybean growth under field conditions (Villafañe et al., 2024). Endophytic *Streptomyces* sp. GMKU 336 significantly supported mung bean growth by increasing elongation and biomass, chlorophyll content, leaf area, leaf color, and adventitious root formation, and it reduced ethylene levels under flooding conditions compared to uninoculated plants (Jaemsaeng et al., 2018).

Salinity poses significant challenges for farmers in soil and crop remediation and management strategies. In Thailand, inland salt-affected soils, resulting from geochemical processes, cover approximately 1.84 million hectares in the northeastern region, while coastal salt-affected soils, derived from seawater, span approximately 0.43 million hectares along the coast. Additionally, 0.063 million hectares are located in other areas (Arunin and Pongwichian, 2015). Eucalyptus has lately emerged as a significant wood species in Thailand due to the annual demand for pulpwood and paper exceeding 3.6 million tons (Manavakun, 2014). *Eucalyptus camaldulensis* Dehn., or the red gum tree, had a deep root system with the majority of the lateral roots located in the upper 28 cm, and it was drought-tolerant. It could grow in saline soil (Sun and Dickinson, 1995). Therefore, *E. camaldulensis* was a prevalent species and suitable for planting in drought and saline areas in Thailand. Recently, there were a few reports of using PGP bacteria to support the growth of eucalyptus in stress conditions. Co-inoculation with *Pseudomonas* strains M25 and N33 substantially supported the growth and photosynthetic capacity of *Eucalyptus grandis* in drought stress conditions (Chaín et al., 2020). *Brevibacterium linens* RS16 promoted growth of *E. grandis* under heat stress by increasing net carbon accumulation and reducing foliar volatiles (Chatterjee et al., 2020). Kaewkla et al. (2025b) reported that *Streptomyces* spp. can support eucalyptus growth in heat, drought, and salinity stress.

Currently, we have studied the biodiversity of endophytic actinobacteria from *E. camaldulensis* grown in a saline area of Kalasin Province, northeastern Thailand, and their potential to inhibit *Eucalyptus* pathogens and to exhibit plant growth-promoting (PGP) traits to support eucalyptus in saline soil (Kaewkla et al., 2025a). This study aimed to identify two *Streptomyces* strains, EKL1.1^T^ and EKS8.28^T^, which were obtained from surface-sterilized red gum trees grown in saline soil, as a novel species using a polyphasic approach. These two strains were investigated for antifungal activity against *Eucalyptus* pathogens and were studied for PGP traits *in vitro* and *in planta*. Moreover, insights into the genomes of these two *Streptomyces* strains were reported.

## Materials and methods

### Isolation of strains EKL1.1^T^ and EKS8.28^T^

Strains EKL1.1^T^ and EKS8.28^T^ were isolated from eucalyptus tissues from the previous study (Kaewkla et al., 2025a). Briefly, the leaves, twigs, and roots of *E. camaldulensis* Denh. (red gum) were collected from saline soil grounds in Ponsim village, Yangtarat district, Kalasin province, Thailand (16.39910°N, 103.27090°E), and surface-sterilized as previously described (Kaewkla et al., 2025a). In previous research, soil salinity at the site where the plant sample was collected was measured by the electrical conductivity of a saturated soil extract (ECe) following the method of Rhoades et al. (1999). Leaf, twig, and root tissues that had been surface-sterilized were spread out across four distinct media (Kaewkla et al., 2025a). Plates were stored for 12 weeks at 27 °C in airtight plastic boxes that were moistened with wet paper towels. Actinobacterial strains were stored in 20% glycerol at −80 °C and on HPDA slants at 4 °C after being purified using half-strength potato dextrose agar (HPDA).

### Phylogenetic analysis of the 16S rRNA gene

Genomic DNA of strains EKL1.1^T^ and EKS8.28^T^ was extracted using a bacterial DNA extraction kit (Vivantis). The amplification of the 16S rRNA gene by polymerase chain reaction (PCR) and sequencing was described in the previous study (Kaewkla and Franco, 2013). 16S rRNA gene sequences of these two strains were analyzed using the EzbioCloud server (Yoon et al., 2017). They were then aligned with the 16S rRNA gene sequences (searched in GenBank/EMBL) of the closely related *Streptomyces* members with validly published names using CLUSTAL X (Thompson et al., 1997), with *Embleya scabrisporus* KM-4927^T^ serving as the out-group. The software program MEGA version 11 (Tamura et al., 2021) was used to construct the phylogenetic trees using the neighbor-joining (NJ) (Saitou and Nei, 1987) and maximum likelihood (ML) (Nei and Kumar, 2000) approaches. To compute ML and NJ estimates, the Tamura-Nei approach (Tamura and Nei, 1993) was used. The tree topology was assessed using a bootstrap approach with 1,000 replications (Felsenstein, 1985).

### Sequencing, assembly, and annotation of genomes

For whole-genome sequencing, a bacterial DNA extraction kit (Vivantis) was used to extract the genomic DNA of strains EKL1.1^T^ and EKS8.28^T^. PCR-free libraries and a small-insert-size library were constructed to sequence the genomes of these two bacteria. An Illumina NovaSeq platform (2 × 150 bp paired-end reads) was used to sequence the samples at the Omics, Chulalongkorn University. The reads were *de novo* assembled using Unicycler (0.4.8) (Wick et al., 2017).

### Genome comparison

Based on the 16S rRNA gene similarity of strains EKL1.1^T^ and EKS8.28^T^, the closest type strains of these two strains, according to the members on the phylogenetic tree of the 16S rRNA gene, were selected for constructing the maximum likelihood (ML) phylogenomic trees. The ML trees were generated using the RAxML algorithm (Stamatakis, 2014) and inferred with the codon tree option in the PATRIC web server^1^ (Wattam et al., 2017). These trees were based on aligned amino acids and nucleotides derived from 628 and 646 single-copy genes for strains EKL1.1^T^ and EKS8.28^T^, respectively, in the genome dataset matched against the PATRIC PGFams database (David et al., 2016). The Type (Strain) Genome Server (TYGS) was used to construct the phylogenomic tree of strains EKL1.1^T^ and EKS8.28^T^ with other closely related type strains corresponding with the members on the 16S rRNA gene phylogenetic tree, with *E. scabrisporus* KM-4927^T^ as the outgroup (Lefort et al., 2015; Meier-Kolthoff and Göker, 2019).

The Genome-to-Genome Distance calculator (GGDC 3.0; BLAST+ technique) was used to compute the digital DNA–DNA hybridization (dDDH) values between strains EKL1.1^T^ and EKS8.28^T^ and their closely related type strains using formula 2 (identities/HSP length) (Meier-Kolthoff et al., 2013; Meier-Kolthoff et al., 2023). The Average Nucleotide Identity (ANI) blast (ANIb) and ANI MUMmer (ANIm) with pairwise genome alignment, between strains EKL1.1^T^ and EKS8.28^T^ and their closely related type strains, were calculated using the ANIb and ANIm algorithms within the JSpecies web service (Richter and Rosselló-Móra, 2009; Richter et al., 2016). CheckM (Parks et al., 2015) assessed the genome quality and contamination of strains EKL1.1^T^ and EKS8.28^T^, and their closely related type strains are shown in Supplementary Table S1.

### Phenotypic characterization

Comparison of genomes indicates that strains EKL1.1^T^ and EKS8.28^T^ represented a distinct species. Based on the 16S rRNA gene similarity, dDDH, ANIb, and ANIm values, the closest type strains to strain EKL1.1^T^ were *S. mexicanus* NRRL B-24196^T^, *Streptomyces thermoviolaceus* subsp. *apingens* JCM 4312^T^, and *Streptomyces cinereospinus* JCM 6917^T^. The closest type strains to strain EKS8.28^T^ were *S. cynarae* JCM 35919^T^, *S. glomeratus* JCM 9091^T^, *Streptomyces chiangmaiensis* TISTR 1981^T^, and *Streptomyces lannensis* TISTR 1982^T^.

A side-by-side comparison was conducted to characterize the phenotypes of the closely related type strains of strains EKL1.1^T^ and EKS8.28^T^. The morphological properties of these strains were examined on eight distinct media: ISP 2, ISP 3, ISP 4, ISP 5, and ISP 7 (ISP; International Streptomyces Project) (Shirling and Gottlieb, 1966; Atlas and Parks, 1993), Bennett’s agar, HPDA, and Nutrient agar (NA) (Atlas and Parks, 1993). ISP 7 was utilized to assess melanin pigment synthesis. Color determination was evaluated according to Centore (2016). Catalase production, assimilation of seven organic acids, and hydrolysis of starch were evaluated following the methodologies outlined in previous studies (Kurup and Schmitt, 1973). The decomposition of *L*-tyrosine, adenine, and hypoxanthine, and acid production from 15 carbohydrates were conducted following previously established procedures (Gordon et al., 1974). The growth at various NaCl concentrations (1, 3, 5, 7, and 10% w/v), temperatures (15, 27, 37, and 45 °C), and pH levels ranging from 4 to 10 (in 1 pH unit increments, with pH adjusted aseptically post-autoclaving) was assessed following a 14-day incubation on ISP 2 medium (Kurup and Schmitt, 1973).

### Chemotaxonomic characterization

The chemotaxonomic characterization of strains EKL1.1^T^ and EKS8.28^T^ was conducted following standard protocols. Whole-cell sugars were examined using the TLC method (Hasegawa et al., 1983), and diaminopimelic acid (DAP) was identified by thin-layer chromatography (TLC) (Bousfield et al., 1985). Cells were cultivated in ISP2 media, washed with sterilized reverse osmosis (RO) water, and freeze-dried. Freeze-dried cells were used to study menaquinone and polar lipids. The extraction and purification of isoprenoid quinones were conducted utilizing the previously established method (Minnikin et al., 1984). The purified menaquinones were examined using reverse-phase liquid chromatography-mass spectrometry (LC–MS) and electrospray ionization mass spectrometry (ESI) with a Shimadzu LCMS-8030 instrument according to the previously described method (Kaewkla et al., 2022).

The polar lipid was examined by two-dimensional TLC as previously outlined using six sprays (Minnikin et al., 1984; Komagata and Suzuki, 1987; Kaewkla et al., 2022). The fatty acid methyl esters (FAMEs) of strains EKL1.1^T^ and EKS8.28^T^, along with their closely related type strains, were examined for FAMEs under identical conditions, adhering to the procedure established by Microbial Identification Inc. (MIDI) (Sasser, 2001). All cultures were incubated for 5 days at 30 °C in Tryptic Soya Broth (Oxoid) in an Erlenmeyer flask at 150 rpm, then collected by centrifugation. Washed cells were freeze-dried, and dried cells were prepared for FAME analysis according to Sasser (2001), with analysis performed using Sherlock software version 6.4.

### Scanning electron microscopy (SEM)

Strains EKL1.1^T^ and EKS8.28^T^ were grown on HPDA at 28 °C for 7 days, and cells and spores were visualized by Scanning Electron Microscopy (the Carl Zeiss version AURIGA). The sample preparation was conducted according to a previously described protocol (Kaewkla and Franco, 2019); however, the samples were dried in the final step using a critical-point dryer (Leica, EM CPD300) and covered with a layer of gold.

### *In vitro* antibacterial and antifungal activity

The bacterium used in the antibacterial bioassay was the plant pathogen, *Ralstonia solanacearum* TISTR 2069, obtained from the Thailand Institute of Scientific and Technological Research (TISTR). The bacterial assay and inhibition evaluation were modified from the dual culture method with the streak method, and the measurement and assessment of the percentage of inhibition followed the previously described methods (Kampapongsa and Kaewkla, 2016). The percentage of inhibition was recorded as follows: strong inhibition (+ 4, ≥ 90%), good inhibition (+ 3, ≥ 75%), moderate inhibition (+ 2, ≥ 50%), weak inhibition (+, ≥ 15%), and no inhibition (0%).

There were three fungal pathogens that caused leaf spot symptoms of eucalyptus: *Colletotrichum* sp. LS10, *Curvularia* sp. LB12, and *Diaporthe* sp. LS7, and one strain that caused root rot of eucalyptus seedlings, *Fusarium* sp. RE1. They were obtained from the Department of Biology, Faculty of Science, Mahasarakham University, Thailand. The fungal assay was carried out using the dual culture method previously described in the protocol (Kampapongsa and Kaewkla, 2016). The evaluation of fungal inhibition as percent inhibition of radial growth: PIRG.

PIRG (%) = (R1-R2 × 100)/R1.

R1 = The radiance of the fungal colony on the control plate.

R2 = The radiance of the fungal colony on the tested plate.

≥75% PIRG = Strong inhibition (++++) 50–74% PIRG = Good inhibition (+++).

25–49% PIRG = Moderate inhibition (++) 1–25% PIRG = Weak inhibition (+).

0% PIRG = No inhibition (−).

## Plant growth promoting tests *in vitro*

### Indole acetic acid (IAA) production

The production of IAA was investigated following the approach previously outlined (Kaewkla et al., 2022). The supernatant was examined for IAA production using the previously outlined procedure with Salkowski’s reagent (Gordon and Weber, 1951). The experiment was conducted in triplicate.

### Phosphate solubilization and 1-aminocyclopropane-1-carboxylic acid (ACC) deaminase production

The ability of strains EKL1.1^T^ and EKS8.28^T^ for phosphate solubilization was investigated according to the standard method, in which NBRIP agar was employed (Castagno et al., 2011; Sukpanoa et al., 2024). The capacity of strains EKL1.1^T^ and EKS8.288^T^ to produce ACC deaminase was evaluated utilizing DF agar (Penrose and Glick, 2003; Kaewkla et al., 2022).

### Nitrogen fixation assay and cellulase production

The nitrogen fixation procedure for strains EKL1.1^T^ and EKS8.28^T^ *in vitro* was conducted using the previously described method by using Nitrogen-free semi-solid (NFb) agar (Baldani et al., 1997; Kaewkla et al., 2022). Additionally, these two strains were evaluated for cellulase production on carboxymethyl cellulose (CMC) agar plates (Islam and Roy, 2018; Kaewkla et al., 2022).

### Growth at different PEG

ISP 2 agar supplemented with polyethylene glycol (PEG) 6,000 at 0, 1, 3, 5, 7, 9, and 10% (W/V) was used for the PEG 6000 test. Cells of strains EKL1.1^T^ and EKS8.28^T^ were streaked in three lines on each agar plate. Growth was observed after incubation at 28 °C for 14 days. Growth was recorded as no growth (−), weak (+), moderate (++), and good growth (+++). The experiment was in triplicate.

## Seed germination tests in salinity conditions

### Optimization of spore inoculum

Strains EKL1.1^T^ and EKS8.28^T^ were evaluated for the seed germination test. Seeds of *E. camaldulensis* were obtained from the Forest Research and Development Office, Royal Forest Department, Thailand. Seeds were surface sterilized according to the method of Kaewkla et al. (2025a). Strains EKL1.1^T^ and EKS8.28^T^ were cultivated on HPDA for 7 days, after which a spore suspension was made in sterilized RO water. The spore suspension was filtered through a sterilized 10-mL syringe containing sterilized cotton to isolate the spores from the mycelia, and a hemocytometer was employed to enumerate the spores at concentrations of 10^6^, 10^7^, and 10^8^ spores/mL. Subsequently, surface-sterilized eucalyptus seeds were immersed in a spore suspension for 1 h, with RO water serving as the control.

Thereafter, 15 inoculation seeds were placed on sterilized Whatman No. 1 paper within a sterilized glass Petri dish (9 cm in diameter) and moistened with 5 mL of sterilized reverse osmosis water. The experiment was conducted in five replicates, with Petri dishes placed in a growth chamber in darkness for 2 days before light exposure. Subsequently, the plates were subjected to cool daylight under a 12/12 light/dark cycle at 28 °C for the next 6 days. Seedlings were assessed for seedling length (SL) (shoot and root lengths) on day 8. The experiment was conducted using a completely randomized design (CRD).

Seeds were recorded daily for germination from days 3 to 8 after the start of the seed germination test. Seedling growth parameters were analyzed with some modifications to a previous study (Zou et al., 2023).


Germination potential(GP%)=(m1/N)×100 (1)

Germination rate(GR%)=(m2/N)×100 (2)

Seedling Length Vigor Index(SLVI)=GR×SL (3)

In the Equation 1, N is the total number of test seeds (*N* = 15) and m1 is the number of normally germinated seeds (root length ≥ 2 mm) within 3 days. In the Equation 2, m2 is the number of normally germinated seeds within 8 days; and In the Equation 3, SL is the average seedling length (cm) after 8 days of germination. Germination potential (GP) in equation 1 followed the previously described protocol (Zou et al., 2023), while germination rate (GR) in Equation 2 and SLVI in Equation 3 were modified by the previous study (Kerbab et al., 2021).

### Seed germination and seedling length vigor index (SLVI) tests in salinity conditions

Based on the result of optimization of spore inoculum for the seed germination test, spore inoculum at 10^7^ and 10^8^ spores/mL was suitable to promote seedling growth of strains EKL1.1^T^ and EKS8.28^T^, respectively. Therefore, the surface-sterilization and spore inoculum methods were performed, as described in the section “Optimization of spore inoculum.” Spore suspensions at 10^7^ and 10^8^ spores/mL were prepared for strains EKL1.1^T^ and EKS8.28^T^, respectively. Inoculated seeds with each bacterium were placed at different concentrations of NaCl (0, 50, 100, 150, and 200 mM). The experiment was conducted in a factorial design (2 factors with 5 levels of salt and two bacterial treatments plus a control treatment without bacteria), and plates were arranged in a completely randomized design (CRD).

Fifteen bacterized seeds were then placed on sterilized Whatman No. 1 paper, lined in a sterilized glass Petri dish (9 cm diameter), and moistened with 5 mL of each salt concentration with sterilized water as a control. Germination potential (GP%), germination rate (GR%), and seedling length vigor index (SLVI) were calculated in the section “Optimization of spore inoculum.”

## Plant growth-promoting *in planta*

### Seed germination

Strains EKL1.1^T^ and EKS8.28^T^ were investigated for their ability to promote the growth of eucalyptus seedlings in soil according to the method of Kaewkla et al. (2025a). The spore suspension of strains EKL1.1^T^ (10^7^ spores/mL) and EKS8.28^T^ (10^8^ spores/mL) was filtered. The seeds of *E. camaldulensis* were prepared and surface sterilized following the previous method in the section “Optimization of spore inoculum.” Surface-sterilized eucalyptus seeds were immersed in a spore suspension of each strain, EKL1.1^T^ or EKS8.28^T^, for 60 min, and seeds were submerged in sterile RO water as a control. A sterilized Petri dish (9 cm in diameter) lined with sterilized Whatman No. 1 filter paper was used to germinate bacterized seeds. The Petri dish contained 20 seeds, moistened with sterilized RO water at pH 7.2, and was maintained at 28 °C in the dark for 4 days.

### Seedling growth

Fine sand was prepared and autoclaved at 121 °C for 30 min twice, as described by Kaewkla et al. (2025a). Plastic trays (17 cm × 26 cm, with 7 × 5 blocks in each tray, each block measuring 3 cm × 3 cm) were used to grow eucalyptus seedlings. Sterilized sand was added to each block. The experiment was performed in triplicate for each treatment (control (water), strains EKL1.1^T^ and EKS8.28^T^). The uniformly grown seedlings cultivated on the Petri dish lined with wetted filter paper in the section “seed germination” were planted at one seedling per block. The experiment was conducted using a Completely Randomized Design (CRD). Plants were maintained in darkness for 2 days before exposure to cool daylight. The planting trays were maintained in a growth chamber under cool daylight conditions (12/12 light/dark cycles) at 28 °C for 14 days. Plants were irrigated with reverse osmosis (RO) water once daily. After 14 days of sowing, the seedlings were carefully removed from the sand, and their roots were thoroughly washed to remove any residual sand. Measurements were taken of seedling lengths (both shoot and root) and the fresh weight of the plants. The dry weight could not be measured after drying the seedlings due to their small size.

### Statistical analysis

IBM SPSS Statistics version 29 (Mahasarakham University License) was applied for statistical analysis. The optimization of the spore inoculum for each actinobacterial strain in the seed germination test was performed using one-way ANOVA. The seed germination test with different salt concentrations used a factorial design with three treatments (two bacteria and without bacteria) and five salt levels. Data were submitted to tests of normality and homogeneity of variance. The statistical analyses were carried out using GLM (General Linear Model) to determine the effectiveness of bacterial seed priming (with bacteria and salt) on the seed germination test. The Tukey method was used to compare means and identify a significant group with a *p*-value of less than 0.05 (*p* < 0.05). For statistical analysis of the PGP test *in planta*, data were tested for normality and homogeneity of variance tests before analysis. One-way ANOVA with the Scheffe method compared the means to identify a significant group, and a *p*-value of less than 0.05 (*p* < 0.05) showed significant differences. The Kruskal–Wallis test was used when the data were not normally distributed.

### Secondary metabolite, pathway, and subsystem pathway prediction

The secondary metabolite analysis shell (antiSMASH) version 8.0 (Blin et al., 2025) was used to predict biosynthetic gene clusters (BGCs) for secondary metabolite synthesis of strains EKL1.1^T^ or EKS8.28^T^. The genomes of strains EKL1.1^T^ and EKS8.28^T^ were annotated by RASTtk (Rong and Huang, 2012) on the Bacterial and Viral Bioinformatics Resource Center (BV-BRC)^2^. The pathways and subsystems of both strains, as derived from the annotation data (PATRIC), were analyzed in a comparative study (Overbeek et al., 2005; Overbeek et al., 2013).

## Results and discussion

### Isolation and ecology of strains EKL1.1^T^ and EKS8.28^T^

Strains EKL1.1^T^ and EKS8.28^T^ were isolated from surface-sterilized tissues of *E. camaldulensis* Dehn. grown in the saline soil. The soil salinity of the plant sample was 12 dS/m (Kaewkla et al., 2025a), which was a strongly saline soil (Abrol et al., 2016). Strain EKL1.1^T^ was isolated from leaf tissue after incubation for 1 week on VL70 plus carboxymethyl cellulose (CMC) (Kaewkla et al., 2025a). Strain EKS8.28^T^ was isolated from twig tissue after incubation for 8 weeks on starch casein nitrate agar (SCNA) (Mohseni et al., 2013; Kaewkla et al., 2025a).

### Cultural and morphological data

**Figure 1:**
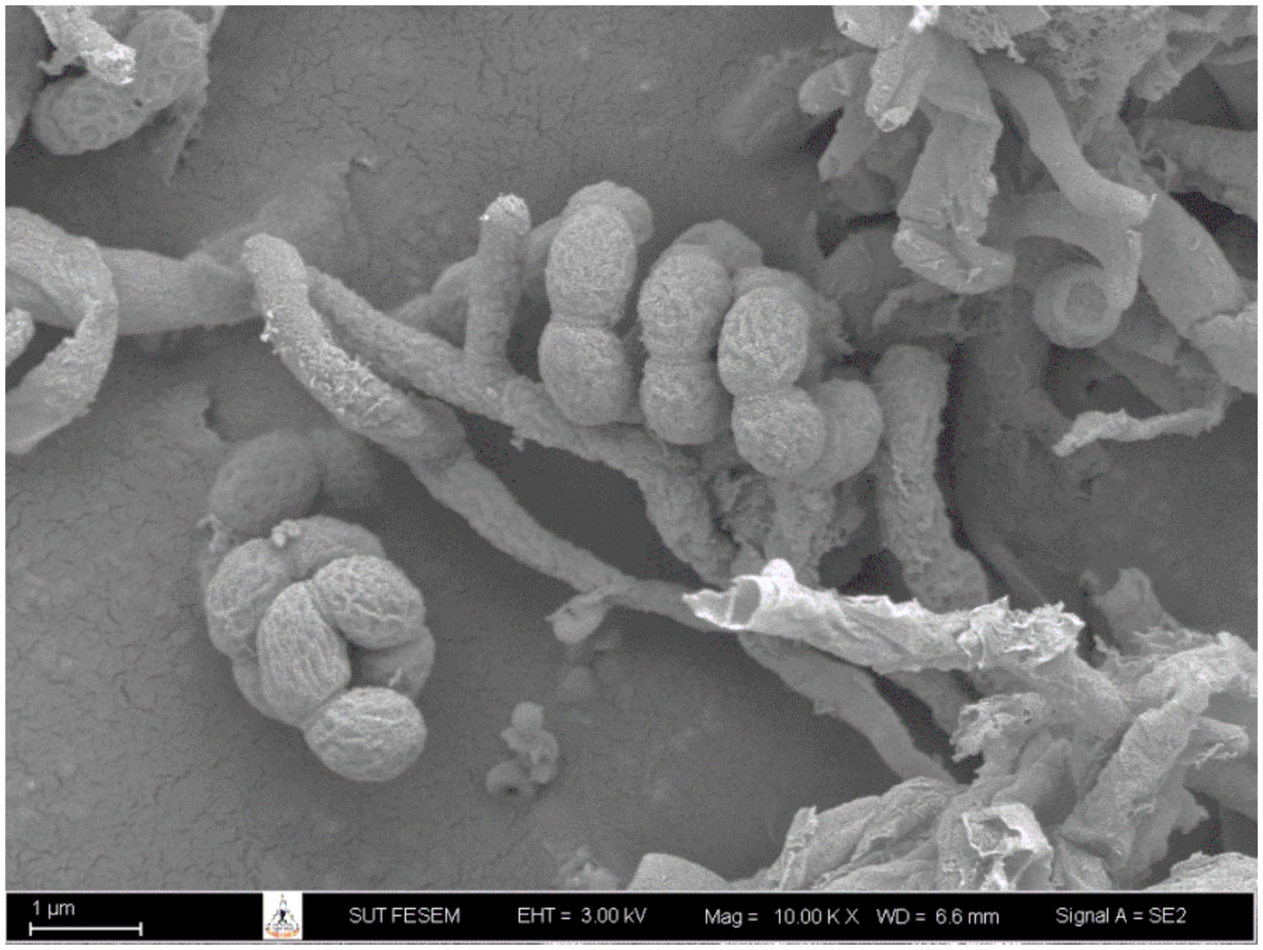
The scanning electron microscope showed hyphae and spores of strain EKL1.1^T^ grown on HPDA for 10 days at 27 °C, with round, rod-shaped spores with warty surfaces and spore chains in spiral ornamental forms. The scale bar represents 1 μm.

The colony morphology of strain EKL1.1^T^ and its closely related type strains was described in Supplementary Table S2. Morphological characteristics of this strain showed well-developed substrate mycelium and aerial mycelia on most media used. No melanin pigment was observed on ISP 7. Strain EKL1.1^T^ formed round rod-shaped spores with warty surfaces and ornamental spores in spiral chains (approximately 1 micron in length × 0.75 micron in diameter) (Figure 1A).

**Figure 2:**
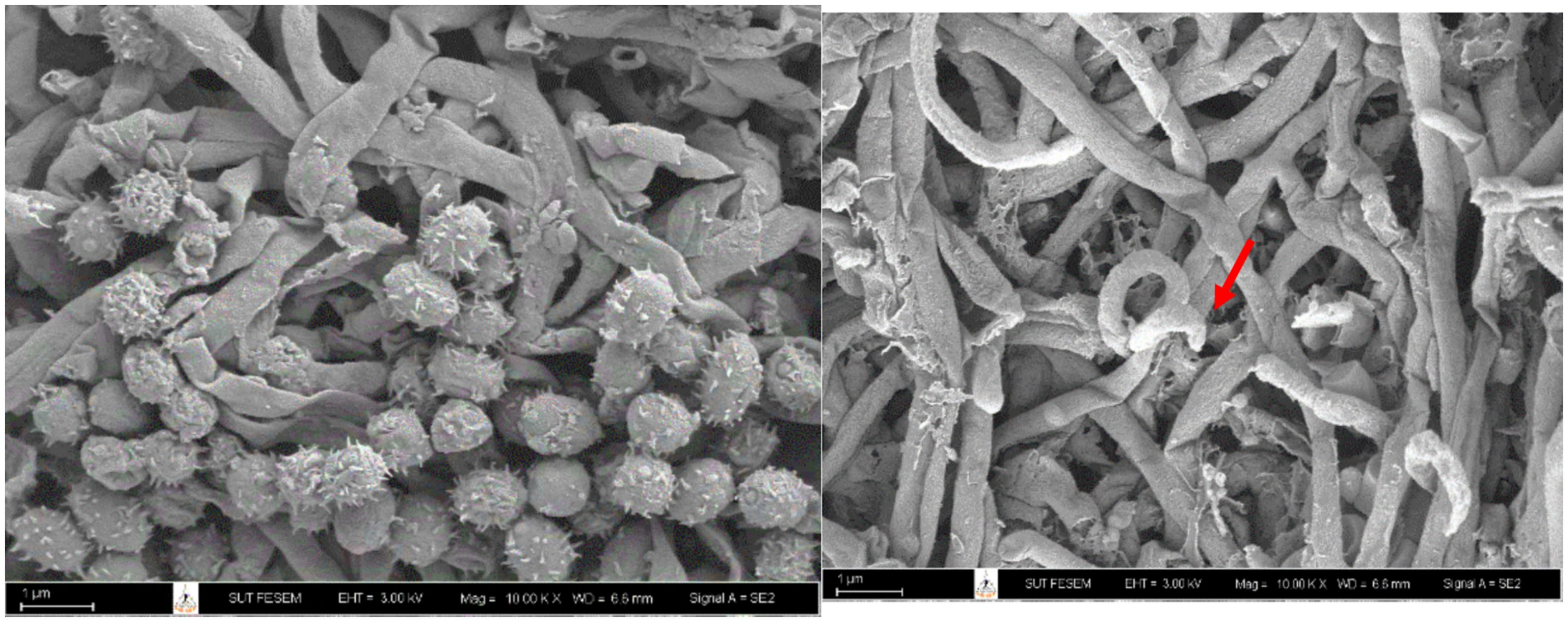
The scanning electron microscope showed hyphae and spores of strain EKS8.28^T^ grown on HPDA for 10 days at 27 °C, with round, rod-shaped spores with spiny surfaces and spore chains in spiral ornamental forms (the arrow indicates spiral chains). The scale bar represents 1 μm.

The colony morphology of strain EKS8.28^T^ and its closely related type strains is described in Supplementary Table S3. Strain EKS8.28^T^ exhibited morphological characteristics with well-developed substrate mycelia and aerial mycelia on most media used. Melanin pigment was observed on ISP 7. Strain EKS8.28^T^ formed oval rod-shaped spores with a spiny surface (approximately 1 micron in length × 0.75 micron in diameter) and spore chains in spiral ornament (Figure 2).

### Chemotaxonomic characteristics

The whole-cell sugars of strains EKL1.1^T^ and EKS8.28^T^ contained galactose and glucose.

Both strains comprised the *LL-*isomer of DAP. The polar lipids of strain EKL1.1^T^ comprised diphosphatidylglycerol (DPG), phosphatidylglycerol (PG), phosphatidylinositol (PI), phospholipid with an amino group (PAG), and one glycolipid (GL) (Supplementary Figure S1A). The polar lipids of strain EKS8.28^T^ contained diphosphatidylglycerol (DPG), phosphatidylglycerol (PG), phosphatidylinositol (PI), phospholipid with an amino group (PAG), three glycolipids (GL), and a lipid with an amino group (LA) (Supplementary Figure S1B). The polar lipid of these two strains was type II (Lechevalier et al., 1977).

The major menaquinones of strain EKL1.1^T^ were MK-9(H_6_) and MK-9(H_8_), while the major menaquinone of strain EKS8.28^T^ comprised MK-9(H_8_).

The major cellular fatty acids (more than 9.5%) of strain EKL1.1^T^ comprised *anteiso*-C_15:0_ (21.8%), *anteiso*-C_17:0_ (21%), *iso*-C_16:0_ (17.2%), and *iso*-C_18:0_ (9.6%), which were similar to the profile of the closest type strain, *S. mexicanus* NRRL B-24196^T^ (Supplementary Table S4). The major fatty acids of the type strain, *S. mexicanus* NRRL B-24196^T^ (≥9.5), were *iso*-C_16:0_ (27.9%), *anteiso*-C_15:0_ (22%), and *anteiso*-C_17:0_ (20.7%). However, cells of the type strain contain *iso*-C_18:0_ at a low level (2.0%) (Supplementary Table S4). Fatty acids of strain EKS8.28^T^ (≥ 9.5%) comprised *anteiso*-C_15:0_ (30.1%) and *iso*-C_15:0_ (27.3%), which were in a similar profile to that of the closest type strain, *S. cynarae* HUAS 13-4^T^. Fatty acids of the type strain contained *anteiso*-C_15:0_ (24.7%), *iso*-C_16:0_ (24.7%), *anteiso*-C_17:0_ (18.5%), and *iso*-C_15:0_ (10.6%), while strain EKS8.28^T^ comprised a lower amount of *anteiso*-C_17:0_ (9.1%) (Supplementary Table S5).

Based on morphological and chemotaxonomic data, these two strains exhibit properties consistent with those of other members of the genus *Streptomyces*.

### 16S rRNA gene analysis and genome comparison between strains EKL1.1^T^ and EKS8.28^T^

The 16S rRNA gene sequences of strains EKL1.1^T^ and EKS8.28^T^ were compared. Strain EKL1.1^T^ shared a 16S rRNA gene similarity of 98.0% with strain EKS8.28^T^. The 16S rRNA gene is a highly conserved gene that was applied to identify prokaryotes at the genus and species level, and the cutoff values at the species level were evaluated between 97.0 and 98.7%, respectively (Stackebrandt and Ebers, 2006). The dDDH, ANIb, and ANIm values between strains EKL1.1^T^ and EKS8.28^T^ were 26, 80.9, and 86.5%, respectively. The species delineation should have ANI values cut off lower than 95–96% (Richter and Rosselló-Móra, 2009), and the dDDH value was lower than the threshold of 70% used to define species level (Chun et al., 2018; Meier-Kolthoff et al., 2023). Therefore, strains EKL1.1^T^ and EKS8.28^T^ were different species.

## Identification of strains EKL1.1^T^ and EKS8.28^T^ as novel species

### 16S rRNA gene analysis and phylogenetic tree of strain EKL1.1^T^

**Figure 3:**
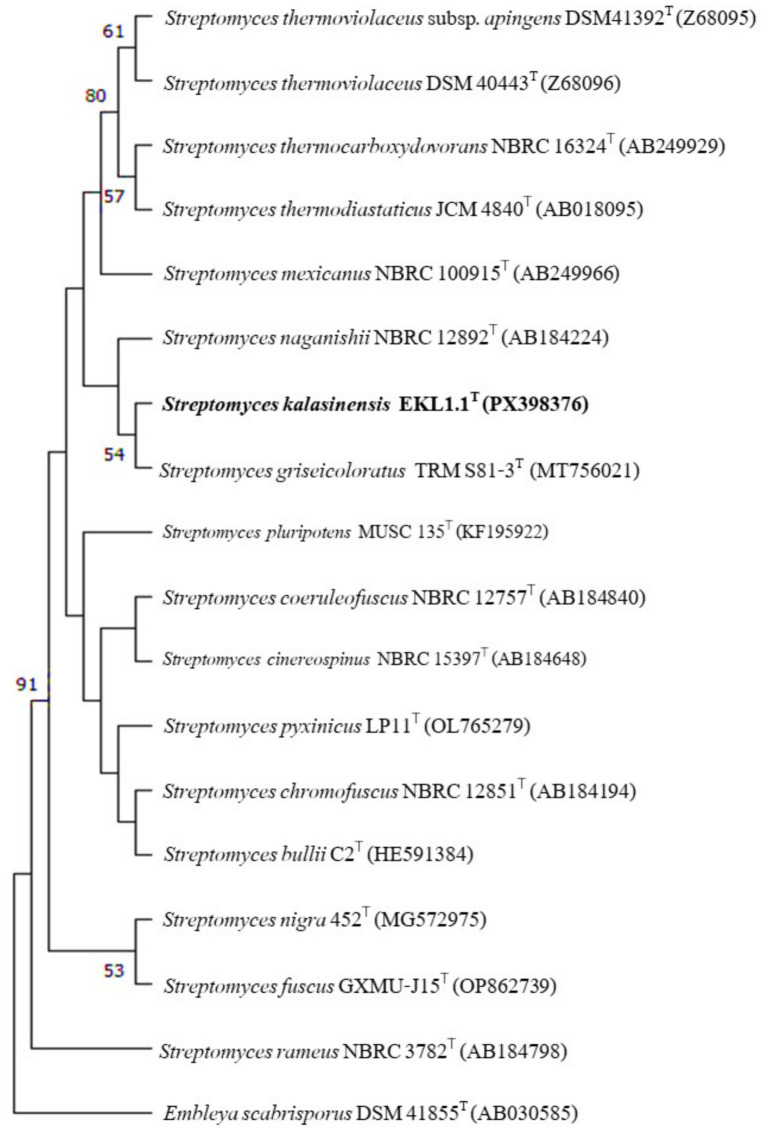
The maximum-parsimony phylogenetic tree is based on 16S rRNA gene sequences (1,360 bp) of *Streptomyces kalasinensis* EKL1.1^T^ and their closely related type strains in the genus *Streptomyces* and *Embleya scabrisporus* KM-4927^T^ as the outgroup. Bootstrap values based on 1,000 replicates are shown at the branch nodes. (The scale bar is not represented, as it is less than 0.000 changes per nucleotide).

The closest type strains, which had the highest 16S rRNA gene sequence similarity with strain EKL1.1^T^, were *S. mexicanus* NRRL B-24196^T^ (99.2%), *S. thermoviolaceus* subsp. *apingens* JCM 4312^T^ (99.1%), and *S. cinereospinus* JCM 6917^T^ (99.1%). However, the closest neighbor on the ML tree, which is positioned in the same clade as strain EKL1.1^T^, was *Streptomyces griseicoloratus* TRM S81-3^T^, with the bootstrap number at 54 (Figure 3). Moreover, the closest neighbors on the NJ tree were *Streptomyces naganishii* NBRC 12892^T^ and *S. griseicoloratus* TRM S81-3^T^, which shared low 16S rRNA gene similarity with strain EKL1.1^T^ at 98.8% and 98.7%, respectively (Supplementary Figure S2).

### Genome comparison of strain EKL1.1^T^

**Figure 4:**
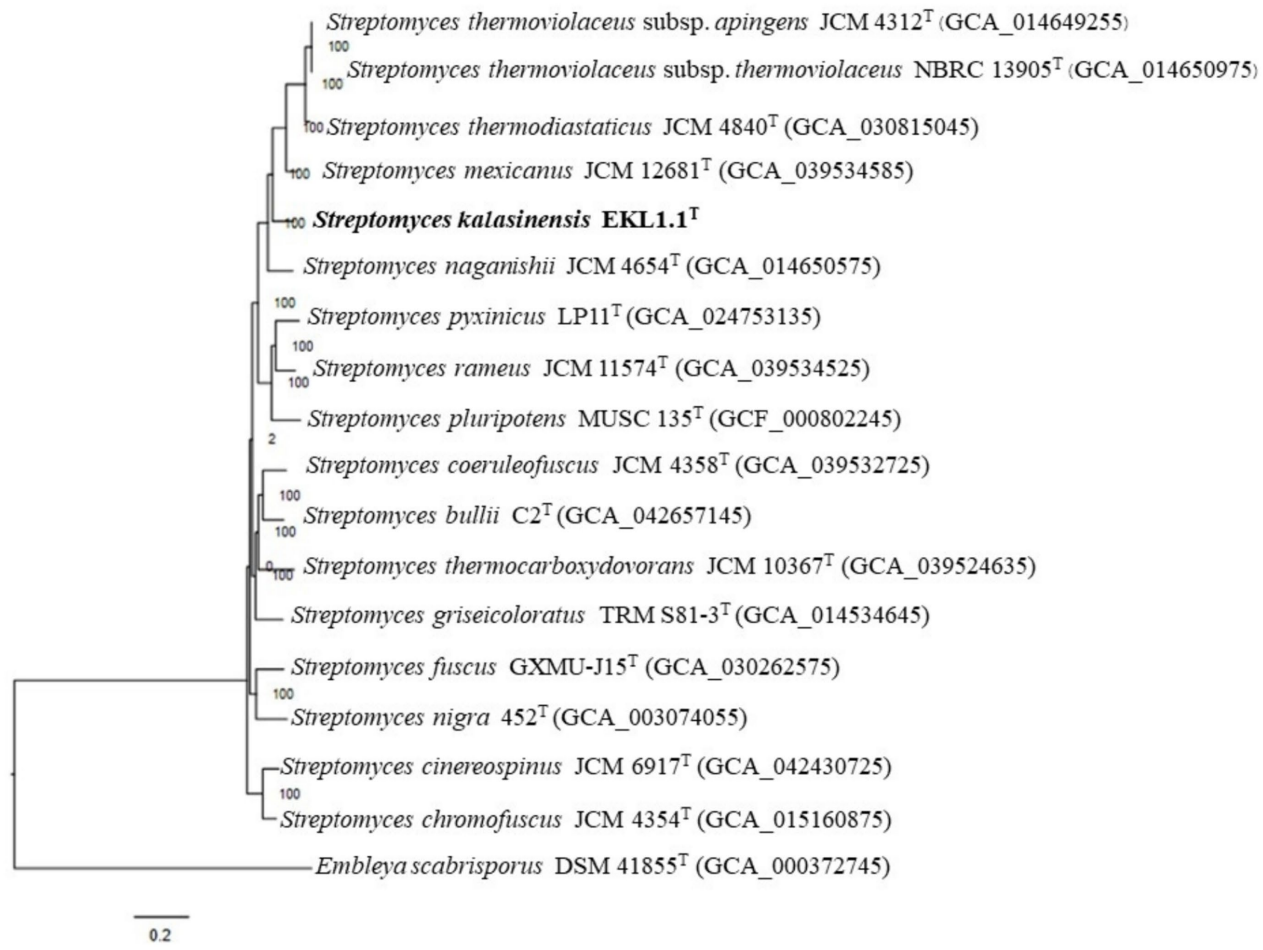
The maximum likelihood (ML) phylogenomic tree using the codon tree option in the PATRIC web server of the genomes of *Streptomyces kalasinensis* EKL1.1^T^ as well as their closely related type strains in the genus *Streptomyces*. *Embleya scabrisporus* KM-4927^T^ as the outgroup. Bar, 0.02 substitutions per nucleotide position.

The draft genome sequence of strain EKL1.1^T^ was 8.09 Mb, with DNA G + C content determined *in silico* as 71.5%, and a total of 7,803 proteins. The genome feature of strain EKL1.1^T^ is presented in Table 1. The ML phylogenomic tree showed the relationship between strain EKL1.1^T^ and the closely related type strains (Figure 4), of which the closest neighbor of strain EKL1.1^T^ was *S. mexicanus* NRRL B-24196^T^ (sharing the highest 16S rRNA gene similarity) (Figure 3). The TYGS phylogenomic tree of strain EKL1.1^T^ and closely related type strains is presented in Supplementary Figure S3. The closest neighbor of strain EKL1.1^T^ is *S. naganishii* NBRC 12892^T^, but they are in different species clusters. Strain EKL1.1^T^ and its closely related species, which shared the highest 16S rRNA gene similarity, *S. mexicanus* NRRL B-24196^T^, had the highest dDDH, ANIb, and ANIm values at 31.8, 85.3, and 88.5%, respectively. The next-closest type strains, which shared the high dDDH, ANIb, and ANIm values with strain EKL1.1^T^, were *S. thermoviolaceus* subsp. *apingens* JCM 4312^T^ at 28.4, 84.3, and 87.4%, respectively (Table 2). However, *S. naganishii* NBRC 12892^T^ and *S. griseicoloratus* TRM S81-3^T^, which were the closest neighbors on the NJ and ML 16S rRNA gene trees, shared lower dDDH and ANIb than *S. mexicanus* NRRL B-24196^T^ (Table 2). Strain EKL1.1^T^ shared dDDH, ANIb, and ANIm values below the species cutoff with the closely related type strains (Stackebrandt and Ebers, 2006; Richter and Rosselló-Móra, 2009; Meier-Kolthoff et al., 2023), with a dDDH value below 70% and an ANI value below 95–96%. Therefore, based on the genome comparison study, strain EKL1.1^T^ was a novel species of the genus *Streptomyces*.

**Table 1:** Genome sequence features of 1, *Streptomyces kalasinensis* EKL1.1^T^, and 2, *Streptomyces phytorum* EKS8.28^T^.

Genome feature	1	2
Genome size (bp)	8,096,726	8,456,457
Number of contigs	238	279
Contig N50	161,277	174,423
Genome completeness	100%	100%
Genome contamination	0.9%	0%
Numbers of Proteins	7,803	8,129
tRNA	73	69
rRNA	5	4
G + C content	71.5%	71.3%
Protein features		
Proteins with functional assignments	5,274	5,398
Proteins with EC number assignments	1,372	1,513
Proteins with GO assignments	1,192	1,326
Proteins with Pathway assignments	1,078	1,173
Proteins with Subsystem assignments	1,506	1,659
Proteins with PATRIC genus-specific family (PLfam) assignments	6,620	6,855
Proteins with PATRIC cross-genus family (PGfam) assignments	6,727	6,956
Specialty Genes (NG)		
Virulence Factor (PATRIC_VF)	3	7
Virulence Factor (Victors)	1	4
Transporter (TCDB)	59	50
Drug Target (DrugBank)	10	9
Drug Target (TTD)	1	1
Antibiotic Resistance (PATRIC)	47	47
Antibiotic Resistance (CARD)	5	7
Antibiotic Resistance (NDARO)	2	2

**Table 2:** 16S rRNA gene pairwise similarity, ANIb, ANIm, AAI, and dDDH values for *Streptomyces* strain EKL1.1^T^ and other closely related type strains in the genus *Streptomyces*.

Strains	Strain EKL1.1^T^	*S. mexicanus* NBRC 100915^T^	*S. pluripotens* MUSC 135^T^	*S. cinereospi-nus* JCM 6917ᵀ	*S. thermovio-laceus* subsp. *apingens* JCM 4312^T^	*S. naganishii* NBRC 12892^T^	*S. griseicolora- tus* TRM S81-3^T^
Strain EKL1.1^T^	*	99.2%, 31.8%, 85.3%, and 88.5%	99.0%, 25.7%, 80.7%, and 86.2%	99.1%, 26.9%, 81.3%, and 86.8%	99.1, 28.4, 84.3%, and 87.4%	98.8, 26.4, 84.5%, and 88.1%	98.7, 27.1, 82.0%, and 86.8%

The order of the list in the table is 16S rRNA gene, dDDH, ANIb, and ANIm values, respectively. * represents 100% for all values.

Based on a genomic comparative study, the four closely related type strains, *S. mexicanus* NRRL B-24196^T^, *S. thermoviolaceus* subsp. *apingens* JCM 4312^T^, *S. cinereospinus* JCM 6917^T^, and *Streptomyces pluripotens* MUSC 135^T^ were selected to study phenotypic differentiation with strain EKL1.1^T^.

### Phenotypic and physiological characterization of strain EKL1.1^T^

The phenotypic properties of strain EKL1.1^T^ and its closely related type strains, *S. mexicanus* NBRC 100915^T^, *S. pluripotens* MUSC 135^T^, *S. cinereospinus* JCM 6917ᵀ, and *S. thermoviolaceus* subsp. *apingens* JCM 4312^T^, were different (Table 3). Strain EKL1.1^T^ was different from the closest type strain, *S. mexicanus* NRRL B-24196^T^, for acid production from *myo*-inositol, rhamnose, and ribose. Strain EKL1.1^T^ could produce acid from these sugars and hydrolyze skim milk and tyrosine, but the type strain could not. On the other hand, *S. mexicanus* NRRL B-24196^T^ could produce acid from maltose and trehalose, but strain EKL1.1^T^ could not. Moreover, the tolerance of sodium chloride of strain EKL1.1^T^ was at 8% NaCl (w/v), while the type strain tolerated salt at 7% NaCl (w/v). Furthermore, the morphology of strain EKL1.1^T^ was different from that of its closest type strain on seven media: ISP 2, ISP 4, ISP 5, ISP 7, Bennet agar, HPDA, and NA (Supplementary Table S2).

**Table 3:** Differential characteristics between *Streptomyces kalasinensis* EKL1.1^T^ and closely related type strains of genus *Streptomyces*.

Charasteristics	1	2	3	4	5
Acid production from:					
arabinose	+	+	−	−	+
maltose	−	+	+	+	+
*myo*-inositol	+	−	−	−	−
mannitol	+	+	−	−	+
rhamnose	+	−	−	−	−
ribose	+	−	−	−	+
trehalose	−	+	−	+	+
xylose	+	+	−	−	+
Acid assimilation:					
lactate	−	−	−	−	+
propionate	+	+	−	−	+
tartrate	−	−	−	−	−
Hydrolysis of:					
esculin	+	+	−	+	+
starch	−	−	+	−	−
skim milk	+	−	+	+	−
adenine	+	+	−	+	+
hypoxanthine	+	+	−	−	+
xanthine	+	+	+	−	−
tyrosine	+	−	+	+	−
Growth at:					
Temperature range (°C)	27–45	27–45	27	27–45	27–45
pH range (pH)	4–10	4–10	5–10	5–10	4–10
Maximum NaCl concentration (% w/v)	8	7	8	3	9

Strains: 1, *Streptomyces kalasinensis* EKL1.1^T^; 2, *Streptomyces mexicanus* NBRC 100915^T^; 3, *Streptomyces pluripotens* MUSC 135^T^; 4, *Streptomyces cinereospinus* JCM 6917ᵀ; and 5, *Streptomyces thermoviolaceus* subsp. *apingens* JCM 4312^T^. All strains could produce acid from cellobiose, fructose, galactose, glucose, and mannose but not from dulcitol, sorbitol, or sucrose. All strains could assimilate acetate and citrate but not benzoate or malate. They could not hydrolyze hippurate or urea. +, positive or present; −, negative or absent.

### Polyphasic study of *Streptomyces* strain EKS8.28^T^

#### 16S rRNA gene analysis and phylogenetic tree of strain EKS8.28^T^

**Figure 5:**
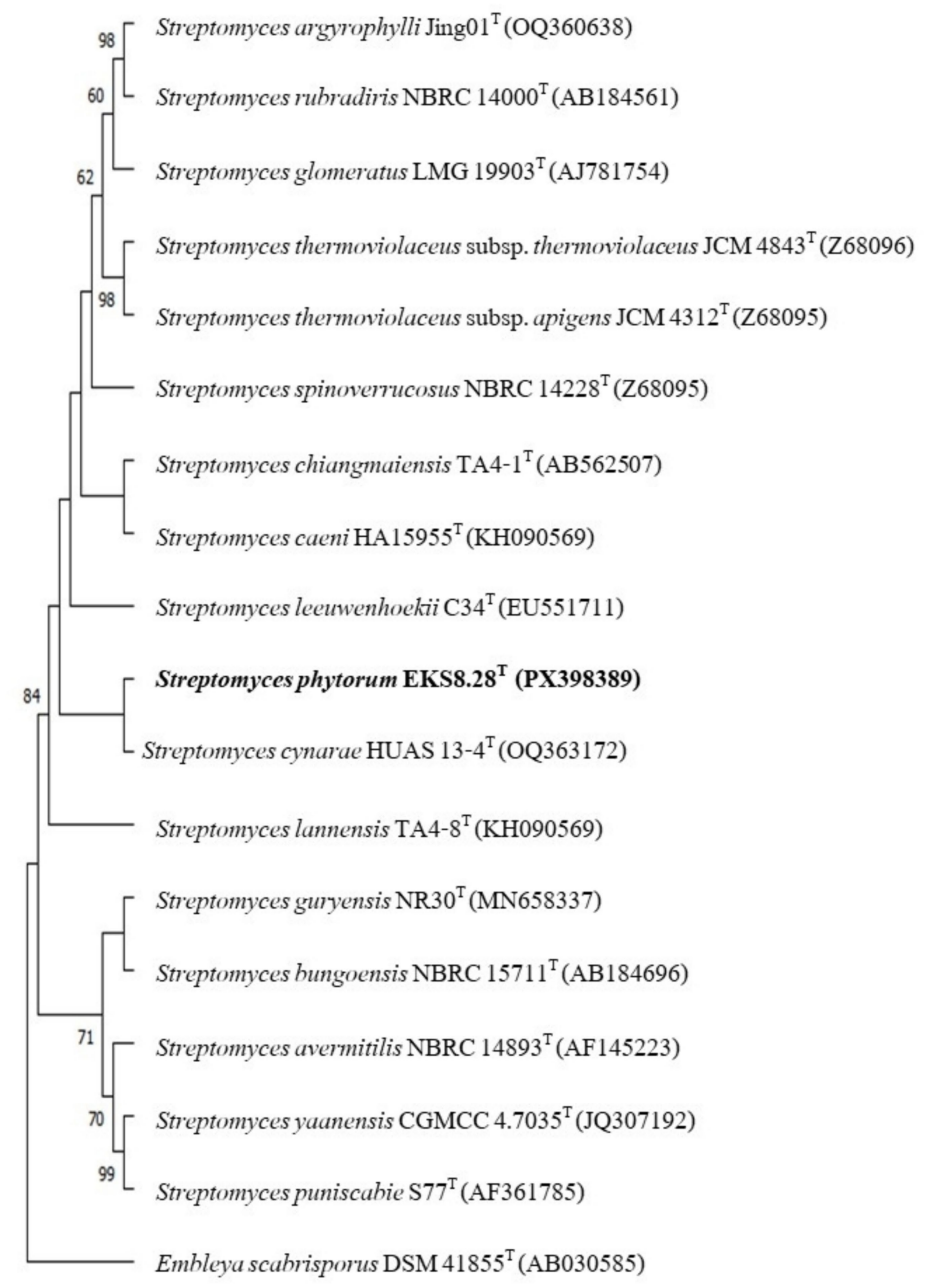
The maximum-parsimony phylogenetic tree is based on 16S rRNA gene sequences (1,356 bp) of *Streptomyces phytorum* EKS8.28^T^ and their closely related type strains in the genus *Streptomyces* and *Embleya scabrisporus* KM-4927^T^ as the outgroup. Bootstrap values based on 1,000 replicates are shown at the branch nodes. (The scale bar is not represented, as it is less than 0.000 changes per nucleotide).

The closest type strains, which had the highest 16S rRNA gene sequence similarity with strain EKS8.28^T^, were *S. glomeratus* LMG 19903^T^ (99.0%), *S. cynarae* HUAS 13-4^T^ (98.9%), *S. chiangmaiensis* TA4-1^T^ (98.9%), and *S. lannensis* TA4-chi8^T^ (98.9%). The closest neighbor on the ML tree, which is positioned in the same clade with strain EKS8.28^T^, was *S. cynarae* HUAS 13-4^T^ (Figure 5). The closest neighbors on the NJ tree were *S. chiangmaiensis* TA4-1^T^ and *Streptomyces caeni* HA15955^T^, which shared 16S rRNA gene similarity with strain EKS8.28^T^ at 98.9 and 98.7%, respectively (Supplementary Figure S4).

### Genome comparison of strain EKS8.28^T^

**Figure 6:**
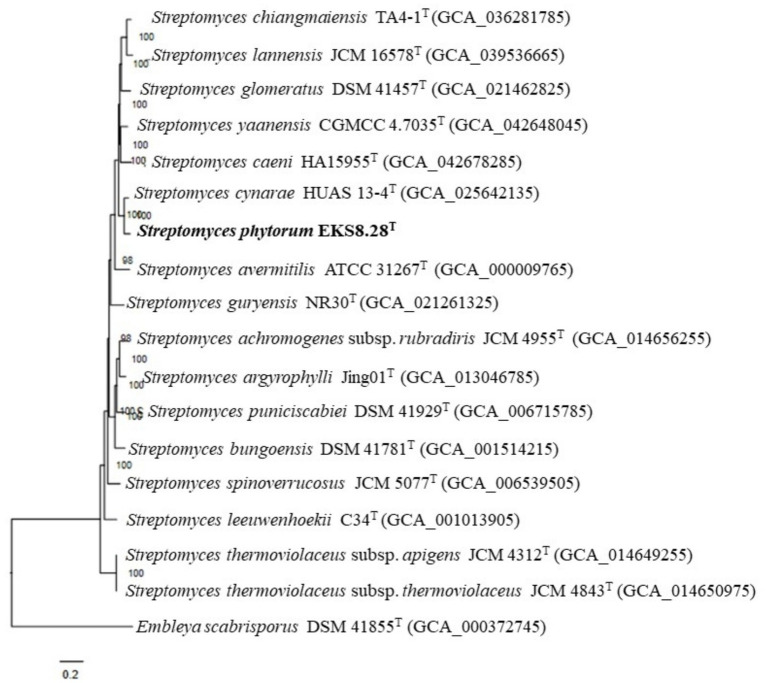
The maximum likelihood (ML) phylogenomic tree using the codon tree option in the PATRIC web server of the genomes of *Streptomyces phytorum* EKS8.28^T^, as well as their closely related type strains in the genus *Streptomyces*. *Embleya scabrisporus* KM-4927^T^ as the outgroup. Bar, 0.02 substitutions per nucleotide position.

The draft genome sequence of strain EKS8.28^T^ was 8.45 Mb, with a DNA G + C content of 71.3% determined by *in silico* analysis, and a total of 8,129 proteins. The genome feature of strain EKS8.28^T^ is presented in Table 1. The ML phylogenomic tree showed the relationship between strain EKS8.28^T^ and the closely related type strains. The ML phylogenomic tree showed that the closest neighbor of strain EKS8.28^T^ was *S. cynarae* HUAS 13-4^T^, which shared the highest 16S rRNA gene similarity (Figure 6). The TYGS phylogenomic tree of strain EKS8.28^T^ and closely related type strains is presented in Supplementary Figure S5. The closest neighbor of strain EKS8.28^T^ on the TYGS phylogenomic tree is *S. cynarae* HUAS 13-4^T^, but they are in a different species cluster.

Strain EKS8.28^T^ and its closely related type strain, *S. cynarae* HUAS 13-4^T^, had the highest dDDH, ANIb, and ANIm values at 41.8, 89.6, and 91.4%, respectively. The next-closest type strain, which shared the high dDDH, ANIb, and ANIm values with strain EKS8.28^T^, was *Streptomyces yaanensis* CGMCC 4.7035^T^ at 31.8, 85.0, and 88.2%, respectively (Table 4). However, *S. yaanensis* CGMCC 4.7035^T^ shared very low 16S rRNA gene similarity with strain EKS8.28^T^ at 96.9%. Strain EKS8.28^T^ had DDH, ANIb, and ANIm values below the species cutoff with the closely related type strains (Stackebrandt and Ebers, 2006; Richter and Rosselló-Móra, 2009; Meier-Kolthoff et al., 2023) (a dDDH value below 70% and an ANI value below 95–96%). A genome-based comparison study showed that strain EKS8.28^T^ was a novel species of the genus *Streptomyces.*

**Table 4:** 16S rRNA gene pairwise similarity, dDDH, ANIb, and ANIm values for *Streptomyces* strain EKS8.28^T^ and other closely related type strains in the genus *Streptomyces*.

Strains	Strain EKS8.28^T^	*S. cynarae* HUAS 13-4^T^	*S. glomeratus* JCM 9091^T^	*S. chiangmaiensis* TISTR 1981^T^	*S. lannensis* TISTR 1982^T^	*S. yaanensis* CGMCC 4.7035^T^
Strain EKS8.28^T^	*	98.9, 41.8, 89.6, and 91.4%.	99.0, 30.3, 84.2, and 87.7%.	98.9, 29.6, 83.6, and 87.4%.	98.9, 28.8, 83.2, and 87.0%.	96.9, 31.8, 85.0, and 88.2%.

The order of the list is 16S rRNA gene, dDDH, ANIb, and ANIm values, respectively. * represents 100% for all values.

Based on genomic analysis, the four type strains, *S. cynarae* HUAS 13-4^T^, *S. glomeratus* JCM 9091^T^, *S. chiangmaiensis* TISTR 1981^T^, and *S. lannensis* TISTR 1982^T^, were selected to study phenotypic differentiation with strain EKS8.28^T^.

### Phenotypic and physiological characterization of strain EKS8.28^T^

The phenotypic properties of strain EKS8.28^T^ and its closely related type strains, *S. cynarae* HUAS 13-4^T^, *S. chiangmaiensis* TA4-1^T^, and *S. lannensis* TA4-8^T^, were different (Table 5). Strain EKS8.28^T^ was different from the closest type strain, *S. cynarae* HUAS 13-4^T^. Strain EKS8.28^T^ produced acid from cellobiose, but the type strain could not. Moreover, strain EKS8.28^T^ could grow at 5% NaCl, but the type strain grew at 7% NaCl. Moreover, the colony morphology of strain EKS8.28^T^ was different from that of *S. cynarae* HUAS 13-4^T^ on ISP 3, ISP 4, ISP 5, ISP 7, Bennett’s agar, HPDA, and NA (Supplementary Table S3). Strain EKS8.28^T^ produced melanin pigment on ISP 7, whereas the type strain could not.

**Table 5:** Differential characteristics between *Streptomyces phytorum* EKS8.28^T^ and closely related type strains of genus *Streptomyces*.

Characteristics	1	2	3	4	5
Acid production from					
arabinose	+	+	−	+	+
cellobiose	+	−	+	+	+
rhamnose	+	+	−	−	+
ribose	+	+	−	−	−
sucrose	+	+	−	−	−
trehalose	+	+	+	−	+
Acid assimilation					
lactate	+	+	−	−	−
Hydrolysis of:					
skim milk	+	+	−	−	+
adenine	+	+	+	−	+
hypoxanthine	+	+	−	−	+
xanthine	−	−	−	−	+
tyrosine	+	+	−	+	−
Growth at					
Temperature range (°C)	27–45	27–45	27–45	27–37	27–45
pH range (pH)	4–10	4–10	5–10	5–10	5–10
Maximum of NaCl concentration (% w/v)	5	7	5	3	5

Strains: 1, *Streptomyces phytorum* EKS8.28^T^; 2, *Streptomyces cynarae* HUAS 13-4^T^; 3, *Streptomyces glomeratus* JCM 9091^T^; 4, *Streptomyces chiangmaiensis* TISTR 1981^T^; and 5, *Streptomyces lannensis* TISTR 1982^T^. Catalase was positive in all strains. All strains produced acid from fructose, galactose, glucose, maltose, mannose, *myo*-inositol, mannitol, and xylose but not from dulcitol or sorbitol. They could assimilate acetate, citrate, and propionate but not benzoate or tartrate. They could not hydrolyze esculin, hippurate, starch, or urea. +, positive or present; −, negative or absent.

Based on the results of the polyphasic study, strains EKL1.1^T^ and EKS8.28^T^ are proposed as representatives of a novel species of the genus *Streptomyces.* Strain EKL1.1^T^ is named *Streptomyces kalasinensis* sp. nov., and strain EKS8.28^T^ is named *Streptomyces phytorum* sp. nov.

### *In vitro* antibacterial and antifungal activities

Strains EKL1.1^T^ and EKS8.28^T^ did not inhibit the bacterial pathogen, *R. solanacearum* TISTR 2069. Strain EKL1.1^T^ could only inhibit one fungus, *Cladosporium* sp. LB1, moderately (35%) (Supplementary Figure S6). Strain EKS8.28^T^ could inhibit *Fusarium* sp. RE1 (71.1%), *Curvularia* sp. LB12 (62.2%), and *Cladosporium* sp. LB1 (50%). Strain EKS8.28^T^ was unable to inhibit *Colletotrichum* sp. LS10 and *Diaporthe* sp. LS7 (Table 6 and Supplementary Figure S6).

**Table 6:** Plant growth-promoting properties of strains EKL1.1^T^ and EKS8.28^T^ and ability to inhibit bacterial and fungal pathogens *in vitro*. 3+, good inhibition 2+, moderate inhibition; −, no inhibition; P, positive result; ng, negative result; ++, moderate growth; +, weak growth.

PGP traits/inhibition	Strain EKL1.1^T^	Strain EKS8.28^T^
Growth at 10% (w/v) PEG 6000	++	+
IAA production (μg/mL)	17.6	10.2
Cellulase production	ng	ng
Phosphate solubilization	P	P
Nitrogen fixation on NFB medium	ng	ng
Inhibition of		
*R. solanacearum* TISTR 2069	−	−
*Fusarium* sp. RE1	−	3 + (71.1%)
*Cladosporium* sp. LB1	2 + (35%)	2 + (50%)
*Colletotrichum* sp. LS10	−	−
*Curvularia* sp. LB12	−	3 + (62.2%)
*Diaporthe* sp. LS7	−	−

### Plant growth-promoting traits *in vitro* and growth at different PEG concentrations

Strains EKL1.1^T^ and EKS8.28^T^ could not produce ACC deaminase, could not fix nitrogen on NFB medium, and could not produce cellulase. Strains EKL1.1^T^ and EKS8.28^T^ could solubilize phosphate (Supplementary Figure S7). Strains EKL1.1^T^ and EKS8.28^T^ produced IAA at 17.6 and 10.2 μg/mL in ISP 2 with 0.2% *L*-tryptophan, respectively.

Both strains could grow at 10% (w/v) PEG 6000, in which strain EKL1.1^T^ grew moderately (++), while strain EKS8.28^T^ grew weakly (+). It was reported that bacterial strains were evaluated for osmotic stress tolerance by growing them in media containing 0, 10, 20, and 30% polyethylene glycol (PEG-6000). *Bacillus* sp. MN-54, *Enterobacter* sp. FD-17, and *Pseudomonas fluorescens*, which were tolerant to PEG-6000, were selected to test plant growth promotion *in planta* with drought stress and resulted in higher photosynthetic activity (25–39%), preservation of leaf water status (14–18%) and pigments (27–32%), and stimulus of antioxidant compounds (28–38%) than no inoculation in water-stressed maize seedlings (Saleem et al., 2001). Therefore, strain EKL1.1^T^ and strain EKS8.28^T^, which tolerated 10% PEG-6000, may have the ability to promote plant growth in drought stress. This plant growth-promoting property requires further study to prove the ability of these strains.

### Spore concentration for the seed germination test

**Figure 7:**
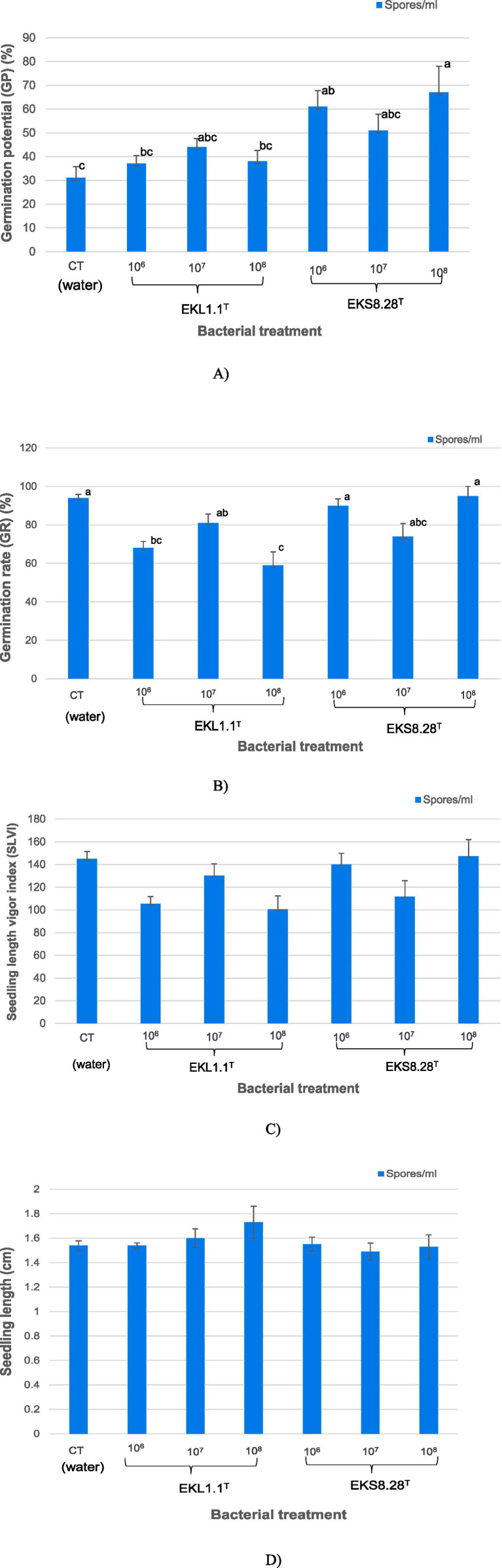
Seed germination test of eucalyptus seedlings treated with control (water), strains EKL1.1^T^ and EKS8.28^T^, at different concentrations of spores at 10^6^, 10^7^, and 10^8^ spores/mL. **(A)** Germination potential, **(B)** germination rate, **(C)** SLVI: seedling length vigor index, **(D)** seedling length. Values represent means ± standard error. One-way ANOVA with Tukey’s Honestly Significant Difference (HSD) test was applied for statistical analysis. The different lowercase letters indicate significant differences (*p <* 0.05).

The optimization of spore concentration for the seed germination test of strains EKL1.1^T^ and EKS8.28^T^ showed that germination potential (GP), germination rate (GR), and seedling length vigor index (SLVI) of treatment EKS8.28^T^ at 10^8^ spores/mL were the highest. GP of strain EKS8.28^T^ at this concentration was significantly higher (*p* < 0.05) than control and treatment EKL1.1^T^ at 10^6^ and 10^8^ spores/mL (Figure 7A). GR of strain EKS8.28^T^ at 10^8^ spores/mL was significantly higher (*p* < 0.05) than treatment EKL1.1^T^ at 10^6^ and 10^8^ spores/mL (Figure 7B).

For treatment EKL1.1^T^, spore concentration at 10^7^ spores/mL was the best to have higher GP, GR, and SLVI than at 10^6^ and 10^8^ spores/mL, and only GR at 10^7^ spores/mL was significantly different (*p* < 0.05) from these two concentrations. However, the GR and SLVI of treatment EKL1.1^T^ at 10^7^ spores/mL were lower than the control, but the difference was not significant (Figure 7C). GP and seedling length (SL) of treatment EKL1.1^T^ at 10^7^ spores/mL were higher than the control but not significantly different (Figure 7D). However, SL in treatment EKL1.1^T^ at 10^8^ spores/mL was the highest, but it was not significantly different from other treatments. Therefore, spore inoculum of strains EKL1.1^T^ and EKS8.28^T^ at 10^7^ and 10^8^ spores/mL, respectively, was used for seed inoculation in a seed germination test in saline conditions. It was reported that the level of spore inoculum of *Streptomyces* sp. had influenced the seed vigor index, in which *Streptomyces* strains JAS2 and TKR8 at 1 × 10^7^ spore/mL and TBS5 at 1 × 10^6^ spore/mL showed the highest vigor index of rice seedlings (Ngalimat et al., 2022).

### Seed germination test in saline conditions

Based on the inoculated seed-germination tests by germinating eucalyptus seeds on filter papers wetted with 0, 50, 100, 150, and 200 mM of sodium chloride (w/v), germination potential (GP), germination rate (GR), seedling length (SL), and seedling length vigor index (SLVI) are shown in Table 7 (Supplementary Figure S8). The results showed that eucalyptus seeds did not germinate at 150 mM at day 3, and seeds did not germinate at 200 mM NaCl on days 3 and 8. The overall results showed that the control had the highest GP, GR, SL, and SLVI. The GR, SL, and SLVI of the control were significantly higher than those of treatments EKL1.1^T^ and EKS8.28^T^ (*p <* 0.05), but the GP of the control was not significantly different from that of these two strains.

**Table 7:** Effects of actinobacteria and salt (NaCl) on the seed germination test of eucalyptus seedlings treated with control (water), strains EKL1.1^T^ and EKS8.28^T^ at different salt concentrations.

Treatment	GP (%)	GR (%)	SL (cm)	SLVI (%)
Bacteria				
Control (water)	18.75 ± 4.86	54.75 ± 7.50^a^	0.705 ± 0.130^a^	54.3 ± 13.63^a^
Strain EKL1.1^T^	14.25 ± 3.27	38.75 ± 5.61^b^	0.587 ± 0.087^b^	30.82 ± 6.97^b^
Strain EKS8.28^T^	17.50 ± 4.07	43.5 ± 6.91^b^	0.562 ± 0.092^b^	33.99 ± 7.42^b^
Salt (NaCl)				
0 mM	41.00 ± 4.20^a^	77.00 ± 3.83^a^	1.30 ± 0.28^a^	103.42 ± 10.20^a^
50 mM	20.00 ± 1.75^b^	65.33 ± 3.46^b^	0.63 ± 0.11^b^	40.65 ± 2.42^b^
100 mM	6.33 ± 1.41^c^	33.00 ± 2.87^c^	0.37 ± 0.06^c^	12.55 ± 1.13^c^
150 mM	0 ± 0^c^	7.33 ± 3.04^d^	0.16 ± 0.15^d^	2.20 ± 0.91^d^
Significant				
Bac	NS	*	*	*
Salt	*	*	*	*
IA	NA	*#*	*#*	*#*

GP: Germination potential; GR: Germination rate; SL: Seedling length; SLVI: Seedling length vigor index. Values represent means ± standard error, and different lowercase letters indicate significant differences (*p* < 0.05). The General Linear Model (GLM) was employed to examine a statistically significant interaction effect, and Tukey’s Honestly Significant Difference (HSD) test was used as a post hoc analysis to assess significant differences among treatments. NS: not significant; IA: interaction; NA: no interaction, *: significant (*p* < 0.05), #: with interaction.

**Figure 8:**
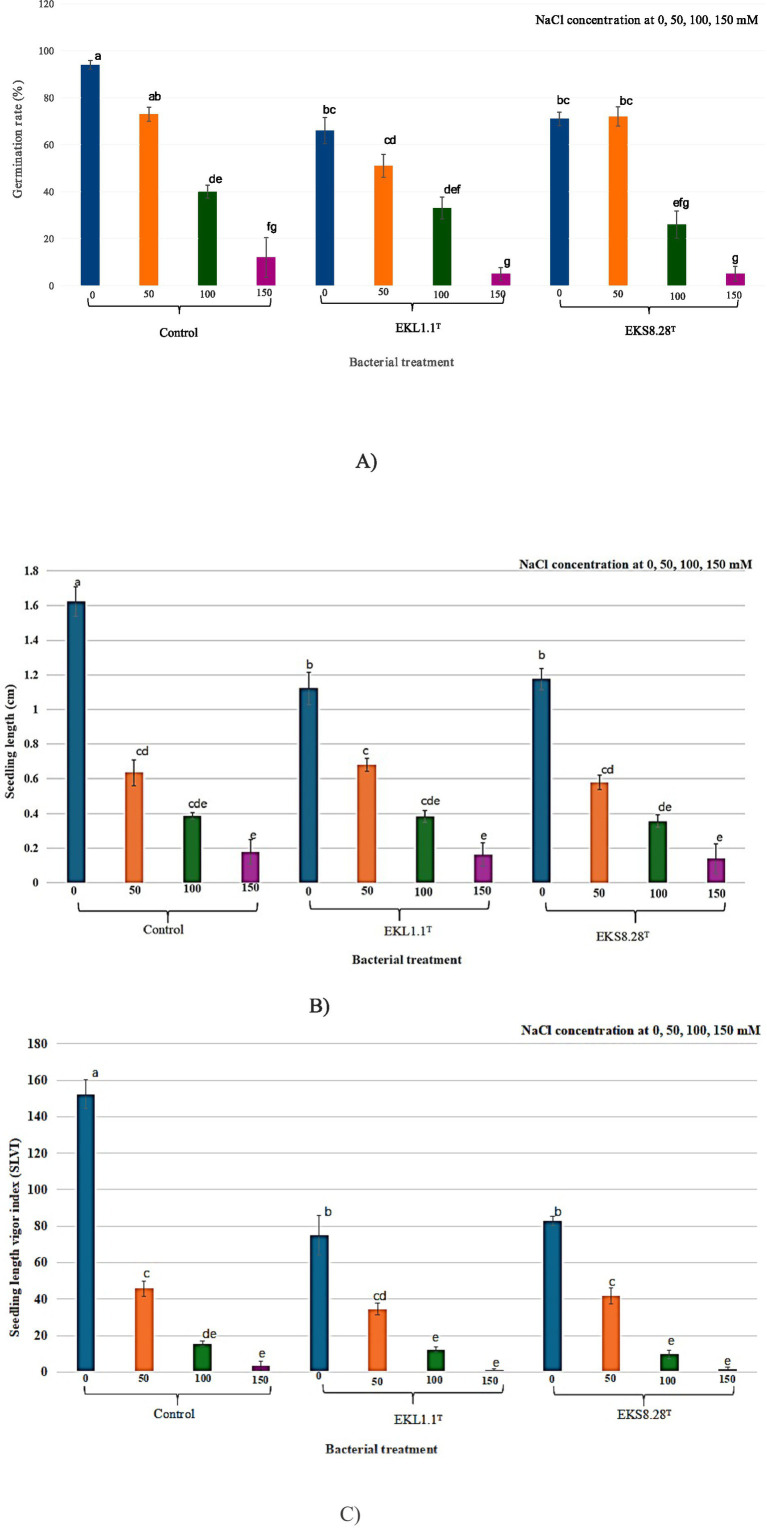
Seed germination test of eucalyptus seedlings treated with control (water), strains EKL1.1^T^, and EKS8.28^T^ at different salt concentrations. **(A)** GR: Germination rate; **(B)** SL: Seedling length; **(C)** SLVI: Seedling length vigor index. Values represent means ± standard error. The general linear model (GLM) was employed to examine a statistically significant interaction effect, and Tukey’s honestly significant difference (HSD) test was used as a *post hoc* analysis to assess significant differences among treatments. There were interactions between bacteria and salt for GR, SL, and SLVI. The different lowercase letters indicate significant differences (*p* < 0.05).

The overall effect of salt on seed germination is shown in Table 7. Concentrations of NaCl at 50, 100, and 150 mM have affected GP, GR, SL, and SLVI by reducing these seed germination parameters. All seedling growth parameters at 50, 100, and 150 mM NaCl were significantly different from those at 0 mM NaCl. There was an interaction between bacterial treatment and salt for GR, SL, and SLVI but not GP (Figure 8).

### The interaction of actinobacteria and salt on germination rate (GR), seedling length (SL), and seedling length vigor index (SLVI) of eucalyptus seedlings

The results showed that at 0 mM NaCl, the GR of seedlings in the control experiment was the highest, which was significantly different (*p* < 0.05) from treatments EKL1.1^T^ and EKS8.28^T^ (Figure 8A). At 50 mM NaCl, the GR of treatment EKS8.28^T^ was a little bit higher than at 0 mM NaCl, whereas the GR of control and treatment EKL1.1^T^ were lower than at 0 mM NaCl. However, they were not significantly lower than at 0 mM NaCl. At 100 mM NaCl, the GR of the control and treatment EKS8.28^T^ were significantly lower than at 50 mM NaCl, while the GR of treatment EKL1.1^T^ at 100 mM NaCl was not significantly lower than at 50 mM NaCl. The results showed that at 0 mM NaCl, seedling length (SL) in the control experiment was the highest, which was significantly different (*p* < 0.05) from treatments EKL1.1^T^ and EKS8.28^T^ (Figure 8B). At 50 mM NaCl, SL of all treatments was similar and not significantly different. Although SL across all treatments did not differ significantly at 100 mM NaCl, the reduction ratio of SL (SL at 0 mM NaCl/SL at 100 mM NaCl) of the control was the highest, at 4.2-fold. The reduction ratios of SL of treatments EKL1.1^T^ and EKS8.28^T^ were 2.92- and 3.30-fold changes, respectively (Figure 8B). Therefore, strains EKL1.1^T^ and EKS8.28^T^ supported eucalyptus seedlings to grow at 100 mM NaCl.

The results showed that at 0 mM NaCl, the seedling length vigor index (SLVI) of seedlings in the control experiment was the highest, which was significantly different (*p* < 0.05) from treatments EKL1.1^T^ and EKS8.28^T^ (Figure 8C). At 50 mM NaCl, SLVI of all treatments was similar and not significantly different. At 100 mM and 150 mM NaCl, the SLVI across all treatments did not differ significantly. However, at 100 mM NaCl, the reduction ratio of SLVI (SLVI of 0 mM NaCl/SLVI of 100 mM NaCl) of the control was the highest, at 9.9-fold changes, compared to treatments EKL1.1^T^ and EKS8.28^T^ at 6.06- and 8.46-fold changes, respectively.

From the result of the seedling growth test, strain EKS8.28^T^ was the best to support GR of eucalyptus seedlings at 50 mM NaCl, while strain EKL1.1^T^ supported seedling growth at 100 mM NaCl. This study was correlated with the application of *Bacillus spizizenii* FMH45 in radish, which increased seedling length, vigor index, and biomass, as well as chlorophyll content, membrane integrity, and phenol peroxidase concentrations, under salinity conditions (Masmoudi et al., 2021). Moreover, *Agrobacterium tumefaciens* (B1), *Bacillus subtilis* (B2), and *Lysinibacillus fusiformis* (B3) improved rice yield by significantly enhancing the developmental traits of rice seedlings under saline stress conditions. Moreover, strain B1 was identified as a potential candidate among the three bacteria, potentially assisting in alleviating salt stress and promoting plant developmental processes (Mahmud et al., 2023).

### Plant growth-promoting *in planta*

**Figure 9:**
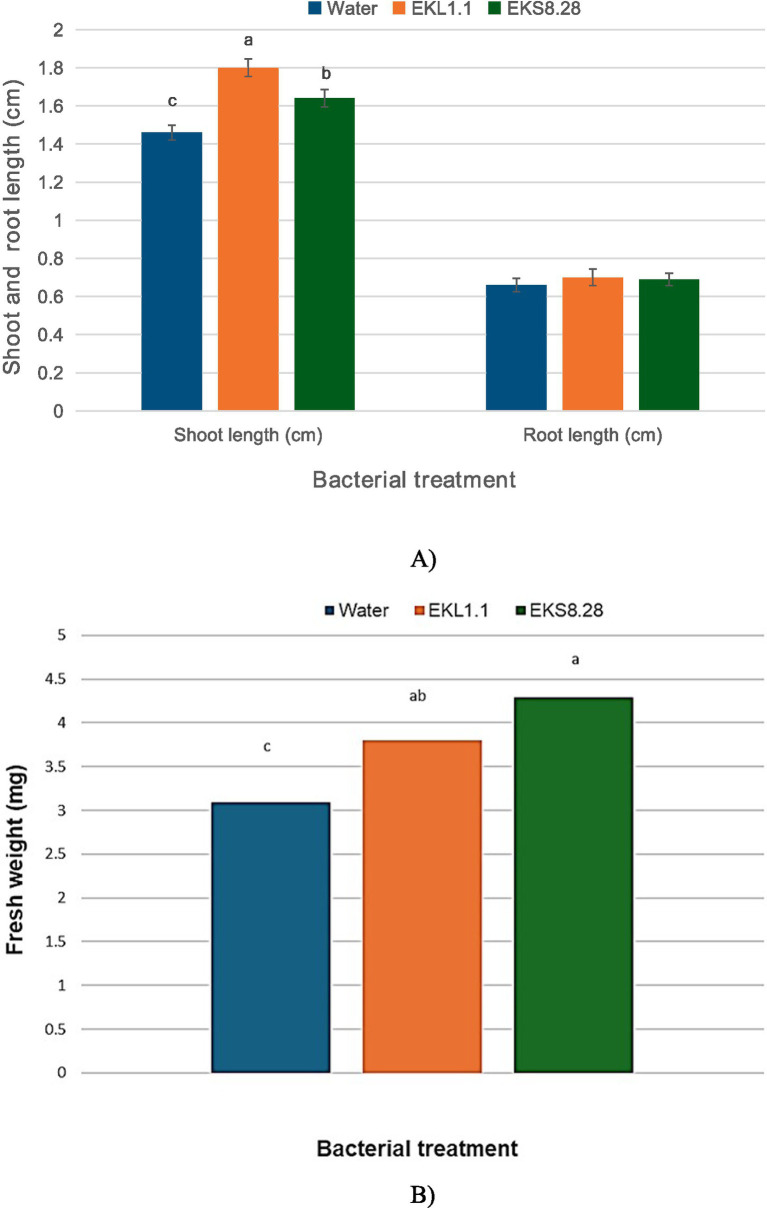
Plant growth-promoting test of eucalyptus seedlings treated with control (water), strains EKL1.1^T^ and EKS8.28^T^, after 14 days of sowing. **(A)** Root length and shoot length; **(B)** Fresh weight. Values represent means ± standard error. One-way ANOVA with the Scheffé test was used for statistical analysis. The different lowercase letters indicate significant differences (*p* < 0.05).

The result of PGP *in planta* of strains EKL1.1^T^ and EKS8.28^T^ is shown in Figures 9 and Supplementary Figure S9. The result showed that the growth of seedlings treated with strains EKL1.1^T^ and EKS8.28^T^ was better than that of the control without actinobacteria to promote eucalyptus seedlings. Shoot length (SL) of treatment EKL1.1^T^ was the highest and significantly different from the control and treatment EKS8.28^T^ (*p* < 0.05). Root length (RL) in treatments EKL1.1^T^ and EKS8.28^T^ was similar and a little bit higher than the control, but they were not significantly different (Figure 9A). The fresh weight of treatment EKS8.28^T^ was the highest and significantly different from the control (*p* < 0.05), but it was not significantly different from strain EKL1.1^T^ (Figure 9B). From the results of the seedling growth test and PGP *in planta*, strain EKS8.28^T^ promoted seed germination, and both strains EKL1.1^T^ and EKS8.28^T^ supported the growth of eucalyptus seedlings. This study was consistent with Niu et al. (2022), who reported that *Streptomyces* strain 11F promotes seed germination and increases switchgrass growth by enhancing rice plant growth and grain yield in greenhouse conditions. Moreover, *Burkholderia phytofirmans* PsJN and biochar were applied together and significantly supported plant growth, grain yield, and nutrient contents of quinoa (Naveed et al., 2020). *Curtobacterium albidum* SRV4 improved rice yield by increasing plant growth parameters, photosynthetic efficiency, and antioxidative enzymatic activities (Vimal et al., 2019).

### Secondary metabolites and biosynthetic gene cluster prediction

*Streptomyces* strains EKL1.1^T^ and EKS8.28^T^ have BGCs for geosmin and hopene, which are common substances in many *Streptomyces* strains (Supplementary Table S6). Furthermore, the genomes of these two strains include spore pigment and albaflavenone. Albaflavenone is a tricyclic sesquiterpene antibiotic that inhibits bacteria and is synthesized by various *Streptomyces* strains (Moody et al., 2011). The BGCs associated with plant growth promotion and stress response are present in the genomes of strains EKL1.1^T^ and EKS8.28^T^. BGCs of ectoine are found in the genomes of strains EKL1.1^T^ and EKS8.28^T^. Ectoine is an osmoregulatory molecule that enables bacteria and plants to withstand drought, thermal, and salinity stress conditions (Thuoc et al., 2019). These BGCs’s detection of these two genomes correlated with their phenotypes. They can tolerate salt and PEG 6000 *in vitro*. Moreover, strains EKL1.1^T^ and EKS8.28^T^ supported eucalyptus seedling growth in salinity conditions. Strains EKL1.1^T^ and EKS8.28^T^ possess BGCs for the siderophores desferrioxamin B and E.

Strain EKL1.1^T^ contains BGCs, 2-methoxy-5-methyl-6-(13-methyltetradecyl)-1,4-benzoquinone, a naturally occurring derivative of benzoquinone, and is likely associated with the *SRS* gene cluster involved in secondary metabolite biosynthesis. This chemical demonstrated efficacy against the Formosan subterranean termite, *Coptotermes formosanus*, indicating possible use in termite control (Mozaina et al., 2008). Moreover, the genome of strain EKL1.1^T^ contains the BGC for flaviolin, also known as 1,3,6,8-tetrahydroxynaphthalene (T4HN), a compound that enables fungi to produce melanin.

Strain EKS8.28^T^ comprised more unique BGCs than strain EKL1.1^T^ did. BGC of stenothricin was detected in the genome of strain EKS8.28^T^. Stenothricin is a peptide antibiotic synthesized by some strains of *Streptomyces* sp. It demonstrates antibacterial efficacy against both Gram-positive and Gram-negative bacteria, encompassing several clinically significant pathogens. It is also significant for the inclusion of atypical amino acids such as CysA and Dpr (Liu et al., 2014).

Strain EKS8.28^T^ has a BGC encoding minimycin, which has been reported to have activity against both Gram-positive and Gram-negative bacteria and to have significant antitumor activity against transplantable tumors (Kusakabe et al., 1972). The BGC of radamycin is identified in the genome of strain EKS8.28^T^, a thiopeptide that exhibits a robust capacity to activate the *tip*A promoter. This molecule can induce the *tip*A promoter without exhibiting detectable antibiotic action (Rodriguez et al., 2002). Moreover, strain EKS8.28^T^ comprises a BGC encoding a nonribosomal peptide synthetase (NRPS) that produces cysteoamide and a new cysteate-containing lactone product. This lactone is assembled from the monomers phenylacetic acid, valine, cysteate, threonine, β-hydroxyleucine, and β-alanine, followed by intramolecular cyclization to form a lactone ring. This compound is not known for its biological activity (Wang et al., 2018). The genome of strain EKS8.28^T^ contains *AMR*f. It was reported that the *amrf* gene cluster positively regulates the initiation of aerial-mycelium formation in *Streptomyces griseus* (Ueda et al., 1993).

Additionally, strain EKS8.28^T^ possessed a BGC for ε-Poly-L-lysine (ε-PL), a microbial peptide used to preserve packaged food. ε-PL is extensively utilized globally for its extensive antibacterial efficacy against Gram-negative and Gram-positive bacteria, yeasts, and molds (Ye et al., 2013). Due to its bacteriostatic, soluble, biodegradable, and non-toxic properties for both humans and the environment, this molecule is in high demand. The recent production of ε-PL from bacteria in the phylum *Actinomycetota* has been costly, priced at approximately 180 USD/kg (Wang et al., 2021). Moreover, the genome data mining of strain EKS8.28^T^ correlated with its phenotype, which showed good inhibition against *Fusarium* sp. RE1 (71.1%), *Curvularia* sp. LB12 (62.2%), and *Cladosporium* sp. LB1 (50%).

### Pathway prediction

The comparative pathways of strains EKL1.1^T^ and EKS8.28^T^ are shown in Supplementary Table S7. These two strains contained similar, predictable pathways, and secondary metabolite biosynthesis accounted for the majority (23 pathways). For biosynthesis of polyketides and nonribosomal peptides, these two strains comprise the same six pathways except for biosynthesis of 12-, 14-, and 16-membered macrolides, which can be detected only in strain EKL1.1^T^. Both strains synthesize siderophore group nonribosomal peptides, which are associated with their plant growth-promoting properties and enhance eucalyptus seedling growth *in planta*. The PGPB can produce and secrete siderophores under suitable conditions, hence enhancing and controlling iron bioavailability. Inoculation of *Lotus japonicus* ecotype Gifu with *Pantoea eucalypti* M91, a PGPB strain that produces pyoverdine-like and pyochelin-like siderophores in alkaline circumstances, modifies the root architecture, leading to a herringbone pattern of root branching (Campestre et al., 2016).

In the secondary metabolic biosynthetic pathway, these two strains share 22 similar pathways except for beta-lactam resistance, which is detected only in strain EKL1.1^T^. The predicted pathways of these two strains showed that both included biosynthetic pathways for secondary metabolites related to pigments, specifically anthocyanins and carotenoids, the main natural pigments originating in plants. Compounds relating to plant compounds, diterpenoid, flavonoid, isoflavonoid, isoquinoline alkaloid, phenylpropanoid, sesquiterpenoid, stilbenoid, diarylheptanoid, gingerol, terpenoid backbone, tropane, piperidine, and pyridine alkaloid biosynthesis pathways were detected in the genomes of strains EKL1.1^T^ and EKS8.28^T^. Flavonoids, isoflavonoids, and terpenoids present antioxidant activity.

Tropane alkaloids (TAs) are normally produced by some plants, and their derivatives of TAs are extensively used as anticholinergic agents, anesthetics, and in the treatment of motion sickness. It was reported that the endophytic fungus *Colletotrichum incarnatum* could produce TAs (Naik et al., 2018). Diterpenoids have pharmacological activity, including anticancer, anti-inflammatory, and immunological modulating properties (González-Trujano et al., 2015). Ginger (*Zingiber officinale* Rosc.) contains substances that can be categorized into three primary groups: volatile oils, gingerols, and diarylheptanoids. Gingerols represent a homologous series of phenolic compounds that includes shogaols, paradols, and gingerone (Semwal et al., 2015). Diarylheptanoids are a class of derivatives characterized by a 1,7-diarylheptane skeleton, encompassing curcuminoids, which are chemicals present in turmeric (Vanucci-Bacqué and Bedos-Belval, 2021). Strains EKL1.1^T^ and EKS8.28^T^ may produce these compounds relating to plant bioactive substances.

Furthermore, strains EKL1.1^T^ and EKS8.28^T^ contained antibiotic production pathways for novobiocin, penicillin, cephalosporin, puromycin, streptomycin, and tetracycline. Moreover, the zeatin (cytokinin derivative) pathway is also detected in the genomes of strains EKL1.1^T^ and EKS8.28^T^, which is related to their phenotypes to support plant growth in salt stress and promote shoot length and fresh weight in eucalyptus seedlings. Cytokinin is a plant hormone primarily known for promoting cell division and differentiation, affecting multiple facets of plant growth and development. It also contributes to the delays of leaf senescence and influences apical dominance (Cortleven et al., 2019). Moreover, foliar spraying with exogenous cytokinin can enhance plant drought resistance (Islam et al., 2021).

The degradable pathways of insecticides were detected in two genomes of strains EKL1.1^T^ and EKS8.28^T^, comprising 1,1,1-Trichloro-2,2-bis(4-chlorophenyl) ethane (DDT) degradation, 1,4-dichlorobenzene, Gamma-hexachlorocyclohexane (*γ*-HCH) or lindane, and 2,4-dichlorobenzoate degradation, which are dangerous insecticides that destroy neurotoxins and cause persistent organic pollutants. Both strains contained herbicidal-degradable pathways for atrazine. Moreover, these two strains comprise predictable pathways for degrading compounds in crude oil, including ethylbenzene. Only the genome of strain EKL1.1^T^ comprised the biphenyl degradation pathway. It was reported that *Streptomyces rimosus* could degrade up to 200 mg/L of deltamethrin, a pesticide disrupting calcium channels (Khajezadeh et al., 2020). Moreover, degradation of the pesticide has been studied using *Streptomyces venezuelae* ACT1, which could hydrolyze organophosphorus pesticides (Naveena et al., 2013). Furthermore, *Streptomyces* sp. strain D3 was able to degrade DDT, 3,3′,4,4′-tetrachlorobiphenyl, and pentachloronitrobenzene (Wang et al., 2017).

### Subsystem prediction

Predicted subsystems in subclasses involving DNA repair, stress response, metabolite damage and its repair or mitigation, and protein folding of strains EKL1.1^T^ and EKS8.28^T^ were reported and presented in Supplementary Table S8. Both strains contain similar subsystems. For the protein folding subclass, three subsystems were identified in both genomes: protein chaperones, GroEL and GroES chaperones, and periplasmic disulfide interchange. GroEL and its cochaperonin GroES have been identified as heat-shock proteins. Under thermal stress, the levels of these proteins increase 5–10 times (Martin et al., 1992). Furthermore, molecular chaperones are critical components of protein folding homeostasis (proteostasis). A sophisticated network of chaperones exists in bacteria, performing roles such as aiding protein folding, inhibiting protein aggregation, and disaggregating aggregated proteins (Kim et al., 2021).

Within the subclass of stress responses to cold, thermal, and drought conditions, two subsystems associated with heat and cold shock were discovered. The heat shock DNAK gene cluster extended subsystem was identified in both genomes of strains EKL1.1^T^ and EKS8.28^T^. DnaK, the primary chaperone and a non-ribosome-binding protein in the bacterial cytoplasm, is an essential element of the heat shock response. The dnaK gene is suggested to prevent intramolecular misfolding, hence promoting the posttranslational folding of multidomain proteins under stress circumstances (Selby et al., 2011). Four subsystems were identified in two genomes: choline absorption and conversion to betaine; ectoine and hydroxyectoine uptake and catabolism, ectoine synthesis, and osmoregulation. Organisms acclimatize to hyperosmotic conditions, such as saline conditions, by accumulating organic osmolytes, or compatible solutes, which help modulate osmotic potential without disrupting normal cellular functions. The solutes include carbohydrates, such as trehalose and inositol; amino acids and their derivatives, such as proline and ectoine; and methylammonium and methylsulfonium compounds, including glycine betaine and dimethyl sulfoniopropionate, facilitating survival in various environments (Kempf and Bremer, 1998).

Four subsystems of the subclass proline and 4-hydroxyproline were identified: proline and 4-hydroxyproline uptake and utilization; proline biosynthesis (for review), proline synthesis, and a hypothetical protein related to proline metabolism found in the genomes of strains EKL1.1^T^ and EKS8.28^T^. Proline has been identified as an amino acid associated with bacteria and plants in saline environments. For the subclass of stress response, there were subsystems related to antioxidant mechanisms: a cluster containing glutathione synthetase, glutathione analogs (mycothiol), glutathione (non-redox reactions), glutathione (redox cycle), protection from reactive oxygen species, ergothioneine biosynthesis, possible stress-related actinobacterial clusters, protection from reactive oxygen species, repair of iron centers, and the universal stress protein (USP) family. The genome of strain EKL1.1^T^ contains all these subsystems, while strain EKS8.28^T^ lacks the glutathione non-redox reactions subsystem. In bacteria, glutathione not only plays a crucial role in maintaining the appropriate oxidation state of protein thiols but also significantly safeguards the cell against low pH, chlorine chemicals, and oxidative and osmotic stressors. Furthermore, glutathione has been identified as a posttranslational regulator of protein function during oxidative stress, acting directly by modifying proteins through glutathionylation (Masip et al., 2006).

Moreover, strain EKL1.1^T^ had higher numbers of gene counts in subsystems of the universal stress protein (USP) family (*N* = 15) than strain EKS8.28^T^ (*N* = 9). The subsystem prediction of stress responds to saline conditions related to the ability of strain EKS8.28^T^, which promotes the germination rate of eucalyptus seedlings at 50 mM NaCl, and strain EKL1.1^T^, which promotes the seedling length and SLVI at 100 mM NaCl, compared to the control without actinobacteria. Moreover, the subsystems of both genomes corresponded with their phenotypes, in which strain EKL1.1^T^ could grow at 8% NaCl and moderately grow at 10% PEG 6000 (w/v), and strain EKS8.28^T^ grew at 5% NaCl and weakly grew at 10% PEG 6000 (w/v).

For the subclass of siderophore production, there were two subsystems detected in the genomes of strains EKL1.1^T^ and EKS8.28^T^. Siderophore desferrioxamine E and ferrous iron transporter EfeUOB were detected in both genomes, while the genome of strain EKL1.1^T^ also contains salmochelin-mediated iron acquisition. The genome’s subsystem prediction pathways of strains EKL1.1^T^ and EKS8.28^T^ correlate with their phenotypes, which support the growth of eucalyptus seedlings, in which strain EKL1.1^T^ promoted eucalyptus seedlings by significantly increasing shoot length, and strain EKS8.28^T^ significantly increased fresh weight higher than the control.

For subclass resistance to antibiotics and toxic compounds, two strains comprise subsystems of many antibiotic resistances. Furthermore, both strains comprise subsystems of copper homeostasis: copper tolerance and fusidic acid resistance.

## Conclusion

In conclusion, strains EKL1.1^T^ and EKS8.28^T^ can be differentiated from other species of the genus *Streptomyces* by ANI and dDDH values, a phylogenetic tree of the genomes, and their phenotypic and chemotaxonomic features. The names *Streptomyces kalasinensis* and *Streptomyces phytorum* are proposed for novel species of strains EKL1.1^T^ and EKS8.28^T^, respectively. Strain EKL1.1^T^ could only inhibit one fungus, *Cladosporium* sp. LB1, moderately, while strain EKS8.28^T^ could inhibit *Fusarium* sp. RE1, *Curvularia* sp. LB12, and *Cladosporium* sp. LB1 at good inhibition. Both strains exhibited PGP traits, as evidenced by phosphate solubilization and IAA production. The level of spore inoculum has influenced both strains in their ability to support or regulate the growth of eucalyptus seedlings. Moreover, strains EKL1.1^T^ and EKS8.28^T^ supported eucalyptus seedling growth in salinity conditions. Also, they promoted eucalyptus seedling growth *in planta* by increasing shoot length and fresh weight. Genome mining correlated with properties of these strains *in vitro*. Their genomes comprised BGCs for bioactive compounds and several genes associated with plant growth promotion under stress conditions. Furthermore, both strains contained versatile genes encoding beneficial enzymes and xenobiotic degradation, which can be used in many industries and to remediate polluted environments for further study. These two strains have the potential to increase eucalyptus growth and suppress fungal pathogens, and they can be developed as inoculum to enhance eucalyptus plantations. This promising ability to enhance growth while simultaneously combating pathogens highlights their role in promoting healthier ecosystems. Further research into their specific mechanisms and applications could lead to significant advancements in sustainable agricultural practices, such as improved crop yields and reduced reliance on chemical pesticides.

## Description of *Streptomyces kalasinensis*

*Streptomyces kalasinensis* (ka.la.sin.en’sis. N.L. masc. adj. *kalasinensis*, pertaining to Kalasin province, Thailand, the source of the plant from which the organism was isolated).

Gram stain-positive, aerobic, and catalase-positive. Cells grow between 27 and 45 °C but grow well at 27 °C. Cells grow between pH 4.0 and 10.0 but grow well between pH 7.0 and pH 8.0. Cells can grow in the presence of 8% (w/v) NaCl. Substrate mycelia develop well on all media used, are yellowish-brown, and produce yellowish-brown spores on all media. This strain does not produce melanin pigments on ISP 7. The mycelium is extensively branched and forms spiral spore chains. A rod-shaped spore (0.75 μm diameter × 1 μm length) is observed with a warty surface. Strain EKL1.1^T^ produces acid from arabinose, cellobiose, fructose, galactose, glucose, mannose, *myo*-inositol, mannitol, rhamnose, ribose, and xylose but not from dulcitol, maltose, sorbitol, sucrose, and trehalose. Cells assimilate acetate, citrate, and propionate but not benzoate, lactate, malate, and tartrate. Cells hydrolyze esculin, skim milk, adenine, hypoxanthine, xanthine, and tyrosine but not hippurate, starch, or urea. Whole cells contain *LL*-diaminopimelic acid in their peptidoglycan and galactose and glucose as whole-cell sugars. Polar lipids are diphosphatidylglycerol (DPG), phosphatidylglycerol (PG), phosphatidylinositol (PI), phospholipid with an amino group (PAG), and one glycolipid (GL). MK-9(H_6_) and MK-9(H_8_) are predominant menaquinones. Major cellular fatty acids are *anteiso*-C_15:0_, *anteiso*-C_17:0_, *iso*-C_16:0_, and *iso*-C_18_. The DNA G + C content of the type strain is 71.5%.

The type strain, EKL1.1^T^ (= NRRL B-65753^T^ = TBRC 19936^T^), is an endophytic actinobacterium isolated from leaf tissue of red gum (*Eucalyptus camaldulensis* Dehn.) grown in Yangtalat district, Kalasin province, Thailand. The GenBank/EMBL/DDBJ accession number for the 16S rRNA gene sequence of strain EKL1.1^T^ is PX398376.

## Description of *Streptomyces phytorum*

*Streptomyces phytorum* (phy.to’rum. Gr. neut. n. *phyton*, plant; N.L. gen. pl. n. *phytorum*, of plants).

Gram stain-positive, aerobic, and catalase-positive. Cells grow between 27 and 45 °C but grow well at 27 °C. Cells grow between pH 4.0 and 10.0 but grow well between pH 7.0 and pH 8.0. Cells can grow in the presence of 5% (w/v) NaCl. Substrate mycelia develop well on all media and are yellowish-brown, producing yellowish-green spores. This strain produces melanin pigments on ISP 7. The mycelium is extensively branched and forms spiral spore chains. A rod-shaped spore (0.75 μm diameter × 1 μm length) is observed with a spiny surface. Cells produce acid from arabinose, cellobiose, fructose, galactose, glucose, maltose, mannose, *myo*-inositol, mannitol, rhamnose, ribose, sucrose, trehalose, and xylose but not from dulcitol and sorbitol. Cells assimilate acetate, citrate, lactate, and propionate but not benzoate, malate, and tartrate. Cells hydrolyze skim milk, adenine, hypoxanthine, and tyrosine but not esculin, hippurate, starch, urea, and xanthine. Whole cells contain *LL*-diaminopimelic acid in their peptidoglycan and galactose and glucose as whole-cell sugars. Polar lipids are diphosphatidylglycerol (DPG), phosphatidylglycerol (PG), phosphatidylinositol (PI), a phospholipid with an amino group (PAG), three glycolipids (GL), and a lipid with an amino group (LA). MK-9(H_8_) is a predominant menaquinone, and major cellular fatty acids are *anteiso*-C_15:0_ and *iso*-C_15:0_. The DNA G + C content of the type strain is 71.3%.

The type strain, EKS8.28^T^ (= NRRL B-65754^T^ = TBRC 19937^T^), is an endophytic actinobacterium isolated from the stem tissue of red gum (*Eucalyptus camaldulensis* Dehn.) grown in the Yangtalat district, Kalasin province, Thailand. The GenBank/EMBL/DDBJ accession number for the 16S rRNA gene sequence of strain EKS8.28^T^ is PX398389.

## Data Availability

The datasets presented in this study can be found in online repositories. The names of the repository/repositories and accession number(s) can be found in the article/Supplementary material. The GenBank/EMBL/DDBJ accession number for the 16S rRNA gene sequences of strains EKL1.1^T^ and EKS8.28^T^ are PX398376, and PX398389, respectively.

## Footnotes

1
http://www.patricbrc.org

2
https://www.bv-brc.org
