# Supplementary material for: Two novel species of genus Streptomyces isolated from eucalyptus tissues grown in saline soil and their potential as plant growth promoters
Source: Front Microbiol. 2026 May 29;17:1819523. doi: 10.3389/fmicb.2026.1819523 (PMC13260306; doi:10.3389/fmicb.2026.1819523)
Supplement: Supplementary file 1 [file Data_Sheet_1.pdf]

## Supplementary Tables

# Two novel species of genus *Streptomyces* isolated from Eucalyptus tissues grown in saline soil and their potential as plant growth promoters

Kawintip Kiakhunthod[1], Chanwit Suriyachadkun[2], Sumalee Chookhampaeng[1], Kewalee Prompiputtanaporn[3], Piriya Klankeo[4], Weerachai Saijuntha[5], Onuma Kaewkla[1,5*]

[1]Department of Biology, Faculty of Science, Mahasarakham University, Maha Sarakham Province, 44150, Thailand

[2]Thailand Bioresource Research Center (TBRC), National Center for Genetic Engineering and Biotechnology, National Science and Technology Development Agency, Klong Luang, Pathum Thani 12120, Thailand

[3]Microscopy section, Laboratory Service Unit (LSU), Suranaree University of Technology, Nakhon Ratchasima Province 30000, Thailand

[4]Omics Science and Bioinformatics Center, Faculty of Science, Chulalongkorn University, Pathumwan, Bangkok 10330

[5]Center of Excellence in Biodiversity Research, Mahasarakham University, Maha Sarakham, 44150, Thailand.

* Corresponding author:
Onuma Kaewkla
Email address: onuma.k@msu.ac.th

**Table S1.** Completeness and contamination of genomes of the type strains used for genome comparison study. Genome assessment was carried out by CheckM (Parks et al., 2025).

| NO. | Type strains | GenBank assembly number | Completeness (%) | Contamination (%) |
|---|---|---|---|---|
| 1 | *Streptomyces mexicanus* CH-M-1035$^T$ | GCA_039534585 | 99.47 | 1.36 |
| 2 | *Streptomyces thermoviolaceus* subsp. apingens DSM 41392 $^T$ | GCA_014649255 | 99.49 | 0.28 |
| 3 | *Streptomyces chromofuscus* NBRC 12851$^T$ | GCA_015160875 | 99.53 | 0.19 |
| 4 | *Streptomyces cinereospinus* NBRC 15397 $^T$ | GCA_042430725 | 98.86 | 1.7 |
| 5 | *Streptomyces pluripotens* MUSC 135$^T$ | GCF_000802245 | 99.4 | 1.26 |
| 6 | *Streptomyces coeruleofuscus* NBRC 12757$^T$ | GCA_039532725 | 100 | 0.38 |
| 7 | *Streptomyces naganishii* NBRC 12892$^T$ | GCA_014650575.1 | 99.24 | 1.52 |
| 8 | *Streptomyces bullii* C2$^T$ | GCA_042657145.1 | 99.87 | 1.61 |
| 9 | *Streptomyces thermocarboxydovorans* DSM 44296$^T$ | GCA_039524635.1 | 99.87 | 2.29 |
| 10 | *Streptomyces griseicoloratus* TRM S81-3$^T$ | GCA_014534645.1 | 100 | 1.85 |
| 11 | *Streptomyces thermoviolaceus* subsp. *thermoviolaceus* DSM 40443$^T$ | GCA_014650975.1 | 99.50 | 0.28 |
| 12 | *Streptomyces thermodiastaticus* DSM 40573$^T$ | GCA_030815045.1 | 100 | 0.19 |
| 13 | *Streptomyces pyxinicus* LP11$^T$ | GCA_024753135.1 | 99.24 | 0.51 |
| 14 | *Streptomyces nigra* 452$^T$ | GCA_003074055.1 | 99.46 | 0.19 |
| 15 | *Streptomyces fuscus* GXMU-J15$^T$ | GCA_030262575.1 | 99.9 | 1.45 |
| 16 | *Streptomyces rameus* LMG 20326$^T$ | GCA_039534525.1 | 99.9 | 0.76 |
| 17 | *Streptomyces glomeratus* LMG 19903$^T$ | GCA_021462825.1 | 100 | 0.82 |
| 18 | *Streptomyces yaanensis* CGMCC 4.7035$^T$ | GCA_042648045.1 | 100 | 0.76 |
| 19 | *Streptomyces cynarae* HUAS 13-4$^T$ | GCA_025642135.1 | 97.44 | 1.74 |
| 20 | *Streptomyces chiangmaiensis* TA4-1$^T$ | GCA_036281785.1 | 99.45 | 1.95 |
| 21 | *Streptomyces lannensis* TA4-8$^T$ | GCA_039536665.1 | 100 | 2.29 |
| 22 | *Streptomyces leeuwenhoekii* C34$^T$ | GCA_001013905.1 | 100 | 1.52 |
| 23 | *Streptomyces caeni* HA15955$^T$ | GCA_042678285.1 | 99.85 | 1.77 |
| 24 | *Streptomyces avermitilis* NBRC 14893$^T$ | GCA_000009765.2 | 99.90 | 1.17 |

| | | | | |
|---|---|---|---|---|
| 25 | *Streptomyces guryensis* NR30$^T$ | GCA_021261325.1 | 99.18 | 2.37 |
| 26 | Streptomyces puniciscabiei DSM 41929$^T$ | GCA_006715785.1 | 99.53 | 0.28 |
| 27 | *Streptomyces spinoverrucosus* NBRC 14228$^T$ | GCA_006539505.1 | 99.90 | 2.08 |
| 28 | *Streptomyces argyrophylli* Jing01$^T$ | GCA_013046785.1 | 99.47 | 0.51 |
| 29 | *Streptomyces bungoensis* DSM 41781$^T$ | GCA_001514215.1 | 99.91 | 1.52 |
| 30 | *Streptomyces rubradiris* JCM 4955$^T$ | GCA_014656255 | 99.91 | 1.84 |
| 31 | *Streptomyces thermoviolaceus subsp. thermoviolaceus* JCM 4843 | GCA_014650975.1 | 99.49 | 0.28 |
| 32 | *Streptomyces thermoviolaceus* subsp. *apingens* JCM 4312 | GCA_014649255 | 99.49 | 0.28 |
| 33 | *Embleya scabrispora* DSM 41855 | GCA_000372745.1 | 95.16 | 5.5 |

**Table S2** Cultural characteristics of *Streptomyces* strain EKL1.1$^T$ and closely related type strains of genus *Streptomyces*.

Strain: 1, *Streptomyces kalasinensis* EKL1.1$^T$; 2, *Streptomyces mexicanus* NBRC 100915$^T$; 3, *Streptomyces pluripotens* MUSC 135$^T$; 4, *Streptomyces cinereospinus* JCM 6917$^T$; and 5, *Streptomyces thermoviolaceus* subsp. *apingens* JCM 4312$^T$.

Colony color was recorded according to the color chart (Centore, 2016).

| Media | Characteristic | 1 | 2 | 3 | 4 | 5 |
|---|---|---|---|---|---|---|
| ISP2 | Growth | Good | Good | Good | Moderate | Moderate |
| | Aerial mycelium | Strong orange yellow | Strong orange yellow | Vivid yellow | Strong orange yellow | Strong orange yellow |
| | Substrate mycelium | Strong orange yellow | Strong orange yellow | Grayish red | Strong orange yellow | Strong orange yellow |
| ISP3 | Growth | Good | Good | Good | Moderate | Moderate |
| | Aerial mycelium | Yellowish white | Light yellow | Vivid yellow | Vivid yellow | Strong orange yellow |
| | Substrate mycelium | Yellowish white | Light yellow | Grayish red | Vivid yellow | Strong orange yellow |
| ISP4 | Growth | Good | Good | Good | Good | Moderate |
| | Aerial mycelium | Moderate olive | Moderate yellowish green | Vivid yellow | Light yellow green | Strong orange yellow |
| | Substrate mycelium | Dark grayish yellow | Dark olive green | Grayish red | Light yellow green | Strong orange yellow |
| ISP5 | Growth | Good | Good | Moderate | Moderate | Moderate |
| | Aerial mycelium | Vivid yellow | Deep greenish yellow | Light yellow | Strong orange yellow | Strong orange yellow |
| | Substrate mycelium | Vivid yellow | Deep greenish yellow | Light yellow | Strong orange yellow | Strong orange yellow |

| Media | Characteristic | 1 | 2 | 3 | 4 | 5 |
|---|---|---|---|---|---|---|
| ISP7 | Growth | Good | Good | Moderate | Moderate | Moderate |
| | Aerial mycelium | Light yellow | Strong orange yellow | Light yellow | Strong orange yellow | Light yellow |
| | Substrate mycelium | Dark grayish yellow | Deep greenish yellow | Light yellow | Strong orange yellow | Light yellow |
| Bennett's agar | Growth | Good | Good | Good | Moderate | Moderate |
| | Aerial mycelium | Vivid yellow | Strong orange yellow | Vivid yellow | Strong orange yellow | Light yellow |
| | Substrate mycelium | Vivid yellow | Strong greenish yellow | Vivid yellow | Strong orange yellow | Light yellow |
| HPDA | Growth | Good | Good | Good | Moderate | Moderate |
| | Aerial mycelium | White cream | Strong orange yellow | Strong orange yellow | Moderate yellowish green | Light yellow |
| | Substrate mycelium | Light brown | Vivid yellow | Vivid yellow | Moderate yellowish green | Light yellow |
| NA | Growth | Good | Good | Good | Moderate | Moderate |
| | Aerial mycelium | Vivid yellow | Moderate yellowish green | Light yellow | Strong orange yellow | Light yellow |
| | Substrate mycelium | Vivid yellow | Moderate yellowish green | Light yellow | Strong orange yellow | Light yellow |

**Table S3** Cultural characteristics of *Streptomyces* strain EKS8.28$^T$ and closely related type strains of genus *Streptomyces*.

Strain 1, *Streptomyces phytorum* EKS8.28$^T$; 2, *Streptomyces cynarae* HUAS 13-4$^T$; 3, *Streptomyces glomeratus* JCM 9091$^T$; 4, *Streptomyces chiangmaiensis* TISTR 1981$^T$; and 5, *Streptomyces lannensis* TISTR 1982$^T$. *, melanin pigment production. Colony color was recorded according to the color chart (Centore, 2016).

| Media | Characteristics | 1 | 2 | 3 | 4 | 5 |
|---|---|---|---|---|---|---|
| ISP2 | Growth | Good | Good | Moderate | Good | Good |
| | Aerial mycelium | Light yellow | Light yellow | Bluish black | Deep greenish yellow | Strong orange yellow |
| | Substrate mycelium | Strong yellowish brown | Light yellow | Brilliant yellow green | Deep greenish yellow | Strong yellowish brown |
| ISP3 | Growth | Good | Good | Good | Good | Good |
| | Aerial mycelium | Light yellow | Moderate yellowish green | Moderate yellowish green | Light yellow | Moderate olive |
| | Substrate mycelium | Light yellow | Moderate yellowish green | Moderate yellowish green | Light yellow | Dark olive brown |
| ISP4 | Growth | Moderate | Good | Moderate | Good | Good |
| | Aerial mycelium | Light yellow | Moderate yellowish green | Brilliant yellow green | Dark olive brown | Moderate yellowish green |
| | Substrate mycelium | Grayish brown | Dark olive brown | Moderate yellowish green | Dark olive brown | Moderate yellowish green |
| ISP5 | Growth | Good | Good | Good | Good | Good |
| | Aerial mycelium | Deep greenish yellow | Light yellow | Moderate yellowish green | Deep greenish yellow | Moderate olive |

| Media | Characteristics | 1 | 2 | 3 | 4 | 5 |
|---|---|---|---|---|---|---|
| | Substrate mycelium | Deep greenish yellow | Light yellow | Moderate yellowish green | Deep greenish yellow | Dark olive brown |
| ISP7 | Growth | Good* | Good | Good | Good | Good |
| | Aerial mycelium | Light yellow | Dark olive brown | Bluish black | Deep greenish yellow | Strong orange yellow |
| | Substrate mycelium | Dark grayish yellow | Dark olive brown | Bluish black | Deep greenish yellow | Dark olive green |
| Bennett's agar | Growth | Moderate | Good | Good | Good | Good |
| | Aerial mycelium | Light orange yellow | Strong orange yellow | Moderate yellowish green | Deep greenish yellow | Strong orange yellow |
| | Substrate mycelium | Vivid yellow | Dark olive brown | Moderate yellowish green | Deep greenish yellow | Deep greenish yellow |
| HPDA | Growth | Good | Good | Moderate | Good | Good |
| | Aerial mycelium | Light yellow | Moderate yellowish green | Moderate yellowish green | Moderate yellowish green | Moderate yellowish green |
| | Substrate mycelium | Light yellow | Strong orange yellow | Moderate yellowish green | Moderate yellowish green | Dark olive green |
| NA | Growth | Good | Good | Good | Moderate | Moderate |
| | Aerial mycelium | Vivid yellow | Moderate yellowish green | Grayish brown | Deep greenish yellow | Moderate yellowish green |
| | Substrate mycelium | Vivid yellow | Dark olive brown | Brownish black | Deep greenish yellow | Dark olive green |

**Table S4.** Whole-cell fatty acid composition (%) of 1, *Streptomyces kalasinensis* EKL1.1$^T$; 2, *Streptomyces mexicanus* NBRC 100915$^T$; 3, *Streptomyces pluripotens* MUSC 135$^T$; 4, *Streptomyces cinereospinus* JCM 6917$^T$; and 5, *Streptomyces thermoviolaceus* subsp. *apingens* JCM 4312$^T$. Only fatty acids detected at more than 0.1 % of the total are presented. -, not detected. Bold presents fatty acids ≥ 9.5%.

| **Fatty acids** | **1** | **2** | **3** | **4** | **5** |
|---|---|---|---|---|---|
| *iso*-$C_{14:0}$ | 1.4 | 3.0 | 3.5 | 0.6 | 1.8 |
| $C_{14:0}$ | - | 0.1 | 0.8 | 0.4 | 0.3 |
| *iso*-$C_{15:0}$ | 8.9 | 7.7 | **10.5** | **15.0** | 4.6 |
| *anteiso*-$C_{15:0}$ | **21.0** | **22.0** | **13.7** | **13.9** | **20.3** |
| w8c-$C_{15:0}$ | - | - | - | - | - |
| w8c-$C_{15:1}$ | - | - | - | - | - |
| w6c-$C_{15:1}$ | - | 0.1 | 0.4 | - | - |
| *iso*H-$C_{16:0}$ | - | - | - | - | - |
| *iso*-$C_{16:0}$ | **17.2** | **27.9** | **34.1** | **9.1** | **31.7** |
| $C_{16:0}$ | 1.4 | 1.6 | 4.8 | 6.8 | 2.1 |
| *iso* H-$C_{16:1}$ | 1.7 | 1.7 | 7.3 | 0.7 | 1.1 |
| *anteiso* w9c-$C_{17:1}$ | 3.4 | 2.9 | 3.3 | 4.3 | 2.0 |
| *iso*-$C_{17:0}$ | 7.3 | 5.6 | 3.2 | **11.2** | 4.2 |
| *anteiso*-$C_{17:0}$ | **21.8** | **20.7** | 6.7 | **20.0** | **27.2** |
| w8c-$C_{17.1}$ | 0.3 | 0.1 | 0.7 | 0.8 | - |
| *cyclo*-$C_{17:0}$ | 0.4 | 0.7 | 1.5 | 0.5 | - |
| $C_{17:0}$ | 1.0 | 0.3 | 0.5 | 0.6 | 0.3 |
| *iso* w5c-$C_{17:1}$ | - | - | - | - | - |
| 2OH-$C_{16:1}$ | - | - | - | - | - |
| *iso* H-$C_{18.1}$ | - | 0.2 | 0.8 | 0.2 | - |
| *iso*-$C_{18:0}$ | **9.6** | 2.0 | 0.5 | 0.5 | 2.1 |
| w9c-$C_{18:1}$ | - | 0.2 | 0.5 | 1.5 | 0.4 |
| $C_{18:0}$ | 0.2 | 0.2 | 0.5 | 0.4 | 0.7 |
| *iso* 3OH-$C_{17:0}$ | - | - | - | - | - |

| Fatty acids | 1 | 2 | 3 | 4 | 5 |
| --- | --- | --- | --- | --- | --- |
| 10-methyl, TBSA-$C_{18.0}$ | - | - | - | - | - |
| $C_{20:0}$ | - | - | - | - | - |

**Table S5.** Whole-cell fatty acid composition (%) of Strain 1, *Streptomyces phytorum* EKS8.28$^T$; 2, *Streptomyces cynarae* HUAS 13-4$^T$; 3, *Streptomyces glomeratus* JCM 9091$^T$; 4, *Streptomyces chiangmaiensis* TISTR 1981$^T$; and 5, *Streptomyces lannensis* TISTR 1982$^T$. Only fatty acids detected at more than 0.1% of the total are presented. -, not detected. Bold presents fatty acids ≥ 9.5%.

| Fatty acids | 1 | 2 | 3 | 4 | 5 |
|---|---|---|---|---|---|
| *iso*-$C_{14:0}$ | 1.4 | 2.2 | 0.7 | 0.9 | 1.0 |
| $C_{14:0}$ | 0.3 | 0.2 | - | - | 0.3 |
| *iso*-$C_{15:0}$ | **27.3** | **10.6** | **12.7** | 6.9 | **12.6** |
| *anteiso*-$C_{15:0}$ | **30.1** | **24.7** | **21.4** | **21.6** | **23.1** |
| w8c-$C_{15:0}$ | - | - | - | - | - |
| w8c-$C_{15:1}$ | - | - | - | - | - |
| w6c-$C_{15:1}$ | - | 0.3 | 0.1 | 0.1 | 0.2 |
| *iso*H-$C_{16:0}$ | - | - | - | - | - |
| *iso*-$C_{16:0}$ | 8.2 | **24.7** | **10.7** | **19.8** | **14.7** |
| $C_{16:0}$ | 5.1 | 1.5 | 1.3 | 0.9 | 3.6 |
| *iso* H-$C_{16:1}$ | 0.5 | 1.9 | 2.1 | 1.6 | 1.0 |
| *anteiso* w9c-$C_{17:1}$ | 2.3 | 2.7 | **9.7** | 4.4 | 1.9 |
| *iso*-$C_{17:0}$ | 6.0 | 5.2 | 6.7 | 5.6 | **10.4** |
| *anteiso*-$C_{17:0}$ | 9.1 | **18.5** | **22.2** | **31.6** | **23.0** |
| w8c-$C_{17.1}$ | 0.3 | 0.6 | 0.5 | 0.5 | 0.4 |
| *cyclo*-$C_{17:0}$ | 1.4 | 0.4 | - | 0.2 | 0.5 |
| $C_{17:0}$ | 0.7 | 0.6 | 0.3 | 0.7 | 1.0 |
| *iso* w5c-$C_{17:1}$ | - | - | - | - | - |
| 2OH-$C_{16:1}$ | - | - | - | - | - |
| *iso* H-$C_{18.1}$ | - | - | 0.2 | 0.3 | - |
| *iso*-$C_{18:0}$ | 0.4 | 0.6 | 0.2 | 1.0 | 0.5 |
| w9c-$C_{18:1}$ | - | 0.2 | 0.4 | 0.3 | 0.4 |
| $C_{18:0}$ | - | 0.2 | 0.1 | 0.1 | 1.3 |
| *iso* 3OH-$C_{17:0}$ | - | - | - | - | - |

| **Fatty acids** | **1** | **2** | **3** | **4** | **5** |
| --- | --- | --- | --- | --- | --- |
| 10-methyl, TBSA-$C_{18.0}$ | - | - | - | - | - |
| $C_{20:0}$ | - | - | - | - | - |

**Table S6.** The distribution of BGCs of *Streptomyces kalasinensis* EKL1.1$^T$ and *Streptomyces phytorum* EKS8.28$^T$ based on "antiSMASH" prediction.

| **Type** | **Product** | **Span (nt)** | **Similarity (%)** | **Product** | **Span (nt)** | **Similarity confidence** |
|---|---|---|---|---|---|---|
| | **EKL1.1$^T$** | | | **EKS8.28$^T$** | | |
| terpene | geosmin | 11,439 - 33,631 | high | geosmin | 69,968 - 92,121 | high |
| terpene | hopene | 129,076 - 147,745 | Medium | hopene | 1 - 18,509 | Medium |
| terpene | albaflavenone | 16,759 - 37,772 | high | albaflavenone | 42,052 - 63,065 | high |
| T2PKS | Spore pigment | 1 - 17,085 | high | Spore pigment | 6,903 - 79,412 | high |
| ectoine | ectoine | 14,263 - 24,667 | high | ectoine | 76,353 - 86,751 | high |
| NI-siderophore | desferrioxaminB/ desferrioxamine E | 207,571- 2 37,340 | high | desferrioxamin B/desferrioxam-ine E | 68,186 - 97,958 | high |
| T3PKS | 2-methoxy-5-methyl-6-(13-methyltetradecyl)-1,4-benzoquinone | 109,518 - 150,684 | high | **Not detected** | | |
| T3PKS | flaviolin/1,3,6,8-tetrahydroxynaphthalene | 40,964 - 82,022 | high | **Not detected** | | |
| NAPAA,NRPS,hydrogen-cyanide | **Not detected** | | | stenothricin | 14,840 - 169,348 | high |
| NAPAA | **Not detected** | | | ε-Poly-L-lysine | 11,501 - 45,385 | High |
| NAPAA,NRPS,NRPS-like,T1PKS,betalactone,hglE-KS | **Not detected** | | | minimycin/indigoidine | 88,835 - 197,369 nt. | high |
| azole-containing-RiPP | **Not detected** | | | radamycin/globimycin | 139,737 - 176,087 | high |
| NRPS,NRPS-like | **Not detected** | | | cysteoamide | 5,795 - 69,095 | Medium |
| lanthipeptide-class-iii | **Not detected** | | | AmfS | 43,150 - 60,825 | high |

**Table S7** The predicted metabolic pathways of genomes of 1; *Streptomyces kalasinensis* EKL1.1$^{T}$ and 2; *Streptomyces phytorum* EKS8.28$^{T}$. Y: present; N: not present. (Yellow highlights show the difference between two genomes).

| Pathway numbers | Pathway | Strains | |
|---|---|---|---|
| | | 1 | 2 |
| | **Biosynthesis of Polyketides and Nonribosomal Peptides** | | |
| 522 | Biosynthesis of 12-, 14- and 16-membered macrolides | N | Y |
| 1051 | Biosynthesis of ansamycins | Y | Y |
| 1053 | Biosynthesis of siderophore group nonribosomal peptides | Y | Y |
| 1056 | Biosynthesis of type II polyketide backbone | Y | Y |
| 1057 | Biosynthesis of type II polyketide products | Y | Y |
| 1055 | Biosynthesis of vancomycin group antibiotics | Y | Y |
| 523 | Polyketide sugar unit biosynthesis | Y | Y |
| | **Biosynthesis of Secondary Metabolites** | | |
| 942 | Anthocyanin biosynthesis | Y | Y |
| 312 | beta-Lactam resistance | Y | N |
| 965 | Betalain biosynthesis | Y | Y |
| 905 | Brassinosteroid biosynthesis | Y | Y |
| 232 | Caffeine metabolism | Y | Y |
| 906 | Carotenoid biosynthesis | Y | Y |
| 904 | Diterpenoid biosynthesis | Y | Y |
| 941 | Flavonoid biosynthesis | Y | Y |
| 981 | Insect hormone biosynthesis | Y | Y |
| 943 | Isoflavonoid biosynthesis | Y | Y |
| 950 | Isoquinoline alkaloid biosynthesis | Y | Y |
| 903 | Limonene and pinene degradation | Y | Y |
| 401 | Novobiocin biosynthesis | Y | Y |
| 311 | Penicillin and cephalosporin biosynthesis | Y | Y |
| 940 | Phenylpropanoid biosynthesis | Y | Y |
| 231 | Puromycin biosynthesis | Y | Y |

| Pathway numbers | Pathway | Strains | |
|---|---|---|---|
| | | 1 | 2 |
| 909 | Sesquiterpenoid biosynthesis | Y | Y |
| 945 | Stilbenoid, diarylheptanoid and gingerol biosynthesis | Y | Y |
| 521 | Streptomycin biosynthesis | Y | Y |
| 900 | Terpenoid backbone biosynthesis | Y | Y |
| 253 | Tetracycline biosynthesis | Y | Y |
| 960 | Tropane, piperidine and pyridine alkaloid biosynthesis | Y | Y |
| 908 | Zeatin biosynthesis | Y | Y |
| | **Energy Metabolism** | | |
| 710 | Carbon fixation in photosynthetic organisms | Y | Y |
| 720 | Methane metabolism | Y | N |
| 680 | Methane metabolism | Y | Y |
| 910 | Nitrogen metabolism | Y | Y |
| 190 | Oxidative phosphorylation | Y | Y |
| 195 | Photosynthesis | Y | Y |
| 920 | Sulfur metabolism | Y | Y |
| 720 | Reductive carboxylate cycle ($CO_2$ fixation) | N | Y |
| | **Glycan Biosynthesis and Metabolism** | | |
| 531 | Glycosaminoglycan degradation | Y | Y |
| 604 | Glycosphingolipid biosynthesis - ganglio series | Y | Y |
| 603 | Glycosphingolipid biosynthesis - globo series | Y | Y |
| 563 | Glycosylphosphatidylinositol(GPI)-anchor biosynthesis | Y | N |
| 513 | High-mannose type N-glycan biosynthesis | Y | Y |
| 540 | Lipopolysaccharide biosynthesis | Y | Y |
| 550 | Peptidoglycan biosynthesis | Y | Y |
| 510 | N-Glycan biosynthesis | N | Y |
| | **Lipid Metabolism** | | |
| 592 | alpha-Linolenic acid metabolism | Y | Y |
| 590 | Arachidonic acid metabolism | Y | Y |

| Pathway numbers | Pathway | Strains | |
|---|---|---|---|
| | | 1 | 2 |
| 1040 | Biosynthesis of unsaturated fatty acids | Y | Y |
| 140 | C21-Steroid hormone metabolism | Y | Y |
| 565 | Ether lipid metabolism | Y | Y |
| 61 | Fatty acid biosynthesis | Y | Y |
| 62 | Fatty acid elongation in mitochondria | Y | Y |
| 71 | Fatty acid metabolism | Y | Y |
| 561 | Glycerolipid metabolism | Y | Y |
| 564 | Glycerophospholipid metabolism | Y | Y |
| 591 | Linoleic acid metabolism | Y | Y |
| 120 | Primary bile acid biosynthesis | Y | Y |
| 600 | Sphingolipid metabolism | Y | Y |
| 73 | Synthesis and degradation of ketone bodies | Y | Y |
| | **Metabolism of Cofactors and Vitamins** | | |
| 780 | Biotin metabolism | Y | Y |
| 790 | Folate biosynthesis | Y | Y |
| 785 | Lipoic acid metabolism | Y | Y |
| 760 | Nicotinate and nicotinamide metabolism | Y | Y |
| 670 | One carbon pool by folate | Y | Y |
| 770 | Pantothenate and CoA biosynthesis | Y | Y |
| 860 | Porphyrin and chlorophyll metabolism | Y | Y |
| 830 | Retinol metabolism | Y | Y |
| 740 | Riboflavin metabolism | Y | Y |
| 730 | Thiamine metabolism | Y | Y |
| 130 | Ubiquinone and other terpenoid-quinone biosynthesis | Y | Y |
| 750 | Vitamin B6 metabolism | Y | Y |
| | **Metabolism of Other Amino Acids** | | |
| 410 | beta-Alanine metabolism | Y | Y |
| 460 | Cyanoamino acid metabolism | Y | Y |

| Pathway numbers | Pathway | Strains | |
|---|---|---|---|
| | | 1 | 2 |
| 473 | D-Alanine metabolism | Y | Y |
| 472 | D-Arginine and D-ornithine metabolism | Y | Y |
| 471 | D-Glutamine and D-glutamate metabolism | Y | Y |
| 480 | Glutathione metabolism | Y | Y |
| 440 | Phosphonate and phosphinate metabolism | Y | Y |
| 450 | Selenoamino acid metabolism | Y | Y |
| 430 | Taurine and hypotaurine metabolism | Y | Y |
| | **Translation** | | |
| 970 | Aminoacyl-tRNA biosynthesis | Y | Y |
| | **Signal Transduction** | | |
| 4150 | mTOR signaling pathway | Y | Y |
| 4070 | Phosphatidylinositol signaling system | Y | Y |
| | **Nucleotide Metabolism** | | |
| 230 | Purine metabolism | Y | Y |
| 240 | Pyrimidine metabolism | Y | Y |
| | **Immune System** | | |
| 4660 | T cell receptor signaling pathway | Y | Y |
| | **Xenobiotics Biodegradation and Metabolism** | | |
| 624 | 1- and 2-Methylnaphthalene degradation | Y | Y |
| 351 | 1,1,1-Trichloro-2,2-bis(4-chlorophenyl)ethane (DDT) degradation | Y | Y |
| 627 | 1,4-Dichlorobenzene degradation | Y | Y |
| 623 | 2,4-Dichlorobenzoate degradation | Y | Y |
| 791 | Atrazine degradation | Y | Y |
| 362 | Benzoate degradation via hydroxylation | Y | Y |
| 621 | Biphenyl degradation | N | Y |
| 363 | Bisphenol A degradation | Y | Y |
| 930 | Caprolactam degradation | Y | Y |
| 982 | Drug metabolism - cytochrome P450 | Y | Y |

| Pathway numbers | Pathway | Strains | |
|---|---|---|---|
| | | 1 | 2 |
| 983 | Drug metabolism - other enzymes | Y | Y |
| 642 | Ethylbenzene degradation | Y | Y |
| 364 | Fluorobenzoate degradation | Y | Y |
| 361 | gamma-Hexachlorocyclohexane degradation | Y | Y |
| 281 | Geraniol degradation | Y | Y |
| 980 | Metabolism of xenobiotics by cytochrome P450 | Y | Y |

**Table S8.** The predicted subsystems of genomes of 1; *Streptomyces kalasinensis* EKL1.1$^{T}$ and 2; *Streptomyces phytorum* EKS8.28$^{T}$. Y: present; N: not present. GC: gene count, RC: role count. (Yellow highlights show the difference between two genomes).

| Subclass/ Subsystem Name | 1 | GC | RC | Active | 2 | GC | RC | Active |
|---|---|---|---|---|---|---|---|---|
| **Programmed Cell Death and Toxin** | | | | | | | | |
| Barnase-barstar complex | Y | 3 | 2 | active | N | N | N | N |
| Murein hydrolase regulation and cell death | Y | 5 | 4 | active | Y | 5 | 4 | active |
| Nucleoside triphosphate pyrophosphohydrolase MazG | Y | 1 | 1 | active | Y | 1 | 1 | active |
| Phd-Doc, YdcE-YdcD toxin-antitoxin (programmed cell death) systems | Y | 2 | 2 | active | Y | 2 | 2 | active |
| Possible new toxin-antitoxin system including DivIC | Y | 2 | 2 | active | Y | 2 | 2 | active |
| Toxin-antitoxin replicon stabilization systems | Y | 1 | 1 | likely | N | N | N | N |
| Toxin-Antitoxin system HigAB | Y | 1 | 1 | likely | Y | 2 | 2 | active |
| **Metabolite damage and its repair or mitigation** | | | | | | | | |
| 2-ketoacid oxidoreductases disambiguation | Y | 2 | 3 | active | Y | 2 | 3 | active |
| 2-oxoglutarate dehydrogenase | Y | 3 | 3 | active | Y | 2 | 3 | active |
| 2-phosphoglycolate salvage | Y | 3 | 3 | active | Y | 3 | 3 | active |
| Anaerobic module of TCA | Y | 5 | 3 | active | Y | 8 | 3 | active |
| Cyanobacterial bypass in the TCA | Y | 4 | 4 | active | Y | 5 | 4 | active |
| Dehydrogenase complexes | Y | 25 | 11 | active | Y | 17 | 9 | active |
| Dihydroxyacetone kinases | Y | 3 | 4 | active | Y | 3 | 4 | active |
| DNA Repair Base Excision | Y | 23 | 11 | active | Y | 23 | 11 | active |

| **Subclass/ Subsystem Name** | **1** | **GC** | **RC** | **Active** | **2** | **GC** | **RC** | **Active** |
|---|---|---|---|---|---|---|---|---|
| DNA repair system including RecA, MutS and a hypothetical protein | Y | 3 | 3 | active | Y | 3 | 3 | active |
| DNA repair, bacterial | Y | 20 | 15 | active | Y | 19 | 15 | active |
| DNA repair, bacterial DinG and relatives | Y | 1 | 1 | active | Y | 1 | 1 | active |
| DNA repair, bacterial photolyase | Y | 1 | 1 | active | Y | 1 | 1 | active |
| DNA repair, bacterial RecBCD pathway | Y | 4 | 3 | active | Y | 4 | 3 | active |
| DNA repair, bacterial RecFOR pathway | Y | 10 | 7 | active | Y | 9 | 7 | active |
| DNA repair, bacterial SbcCD exonuclease | Y | 2 | 2 | active | Y | 4 | 2 | active |
| DNA repair, bacterial UvrD and related helicases | Y | 2 | 2 | active | Y | 2 | 2 | active |
| DNA repair, UvrABC system | Y | 5 | 4 | active | Y | 5 | 4 | active |
| Entner-Doudoroff Pathway | Y | 20 | 12 | active | Y | 20 | 12 | active |
| Glycolate, glyoxylate interconversions | Y | 1 | 1 | active | Y | 1 | 1 | active |
| Glycolysis and Gluconeogenesis | Y | 24 | 15 | active | Y | 22 | 16 | active |
| Methylglyoxal Metabolism | N | N | N | N | Y | 8 | 3 | likely |
| Pentose phosphate pathway | Y | 19 | 10 | active | Y | 20 | 12 | active |
| Pyruvate Alanine Serine Interconversions | Y | 8 | 8 | active | Y | 7 | 8 | active |
| Pyruvate metabolism I: anaplerotic reactions, PEP | Y | 14 | 8 | active | Y | 9 | 7 | active |
| Pyruvate metabolism II: acetyl-CoA, acetogenesis from pyruvate | Y | 20 | 8 | active | Y | 19 | 8 | active |
| RecA and RecX | Y | 2 | 2 | active | Y | 2 | 2 | active |
| TCA Cycle | Y | 22 | 14 | active | Y | 32 | 15 | active |
| **Protien folding** | | | | | | | | |

| Subclass/ Subsystem Name | 1 | GC | RC | Active | 2 | GC | RC | Active |
|---|---|---|---|---|---|---|---|---|
| Chaperones GroEL GroES and Thermosome | Y | 12 | 6 | active | Y | 13 | 6 | active |
| Periplasmic disulfide interchange | Y | 1 | 1 | likely | Y | 1 | 1 | Likely |
| Protein chaperones | Y | 13 | 7 | active | Y | 14 | 7 | active |
| **Host-pathogen interactions** | | | | | | | | |
| Hydrolysis of sphingomyelin | Y | 2 | 2 | active | Y | 3 | 2 | active |
| **Stress Response** | | | | | | | | |
| Cluster containing Glutathione synthetase | Y | 3 | 3 | likely | Y | 3 | 3 | likely |
| Ergothioneine biosynthesis | Y | 4 | 4 | active | Y | 6 | 4 | active |
| Glutathione analogs: mycothiol | Y | 12 | 10 | active | Y | 9 | 10 | active |
| Glutathione: Non-redox reactions | N | N | N | N | Y | 3 | 2 | likely |
| Glutathione: Redox cycle | Y | 1 | 1 | active | Y | 1 | 1 | active |
| Possible stress related actinobacterial cluster | Y | 2 | 2 | likely | Y | 2 | 2 | likely |
| Protection from Reactive Oxygen Species | Y | 2 | 2 | active | Y | 6 | 3 | active |
| Repair of Iron Centers | Y | 3 | 1 | likely | Y | 2 | 1 | likely |
| Universal stress protein family | Y | 9 | 1 | active | Y | 15 | 1 | active |
| **Stress Response: Osmotic stress** | | | | | | | | |
| Choline uptake and conversion to betaine clusters | Y | 17 | 8 | active | Y | 21 | 8 | active |
| Ectoine synthesis | Y | 7 | 5 | active | Y | 7 | 5 | active |
| Ectoine, hydroxyectoine uptake and catabolism | Y | 4 | 4 | likely | Y | 4 | 4 | likely |
| Osmoregulation | Y | 3 | 2 | active | Y | 2 | 1 | active |
| **Stress Response: Heat/cold shock** | | | | | | | | |

| Subclass/ Subsystem Name | 1 | GC | RC | Active | 2 | GC | RC | Active |
|---|---|---|---|---|---|---|---|---|
| Cold shock proteins of CSP family | Y | 6 | 2 | active | Y | 7 | 2 | active |
| Heat shock dnaK gene cluster extended | Y | 18 | 13 | active | Y | 19 | 13 | active |
| **Resistance to antibiotics and toxic compounds** | | | | | | | | |
| Actinobacterial signal transduction system MtrAB-LpqB | N | N | N | N | Y | 3 | 3 | active |
| Aminoglycoside modifying enzymes: N-acetyltransferases | Y | 2 | 2 | active | Y | 1 | 1 | likely |
| Aminoglycoside modifying enzymes: O-phosphotransferases | N | N | N | N | Y | 1 | 1 | active |
| Antibiotic targets in cell wall biosynthesis | Y | 4 | 3 | active | Y | 4 | 3 | active |
| Antibiotic targets in DNA processing | Y | 4 | 4 | active | Y | 4 | 4 | active |
| Antibiotic targets in metabolic pathways | N | N | N | N | Y | 7 | 5 | active |
| Antibiotic targets in protein synthesis | N | N | N | N | Y | 10 | 8 | active |
| Antibiotic targets in transcription | Y | 4 | 3 | active | Y | 4 | 3 | active |
| Chloramphenicol resistance | N | N | N | N | Y | 1 | 1 | active |
| Copper homeostasis: copper tolerance | Y | 2 | 2 | active | Y | 2 | 2 | active |
| Fusidic acid resistance | Y | 2 | 2 | likely | Y | 2 | 2 | likely |
| Inner membrane proteins of MarC family, not involved in antibiotic resistance | Y | 1 | 1 | active | Y | 1 | 1 | active |
| Mupirocin resistance | Y | 1 | 1 | likely | Y | 1 | 1 | likely |
| Resistance to Capreomycin and Viomycin | Y | 4 | 4 | active | Y | 4 | 4 | active |

| Subclass/ Subsystem Name | 1 | GC | RC | Active | 2 | GC | RC | Active |
|---|---|---|---|---|---|---|---|---|
| Resistance to Daptomycin | N | N | N | N | Y | 8 | 3 | active |
| Resistance to Triclosan | N | N | N | N | Y | 1 | 1 | active |
| Resistance to Vancomycin and Teicoplanin | N | N | N | N | Y | 3 | 3 | active |
| Tetracycline resistance, all mechanisms | N | N | N | N | Y | 2 | 2 | active |
| **Siderophores** | | | | | | | | |
| Salmochelin-mediated Iron Acquisition | N | N | N | N | Y | 1 | 1 | active |
| Siderophore Desferrioxamine E | Y | 6 | 6 | active | Y | 6 | 6 | active |
| **Proline and 4-hydroxyproline** | | | | | | | | |
| A Hypothetical Protein Related to Proline Metabolism | Y | 3 | 3 | active | Y | 3 | 3 | active |
| Proline biosynthesis (for review) | Y | 12 | 12 | active | Y | 11 | 12 | active |
| Proline Synthesis | Y | 3 | 3 | active | Y | 4 | 4 | active |
| Proline, 4-hydroxyproline uptake and utilization | Y | 9 | 8 | active | Y | 8 | 5 | active |
