# Supplementary material for: Two novel species of genus Streptomyces isolated from eucalyptus tissues grown in saline soil and their potential as plant growth promoters
Source: Front Microbiol. 2026 May 29;17:1819523. doi: 10.3389/fmicb.2026.1819523 (PMC13260306; doi:10.3389/fmicb.2026.1819523)
Supplement: Supplementary file 2 [file Data_Sheet_2.pdf]

Supplementary Figures

Two novel species of genus *Streptomyces* isolated from Eucalyptus tissues grown in saline soil and their potential as plant growth promoters

Kawintip Kiakhunthod[1], Chanwit Suriyachadkun[2], Sumalee Chookhampaeng[1], Kewalee Prompiputtanaporn[3], Piriya Klankeo[4], Weerachai Saijuntha[5], Onuma Kaewkla[1,5*]

[1]Department of Biology, Faculty of Science, Mahasarakham University, Maha Sarakham Province, 44150, Thailand

[2]Thailand Bioresource Research Center (TBRC), National Center for Genetic Engineering and Biotechnology, National Science and Technology Development Agency, Klong Luang, Pathum Thani 12120, Thailand

[3]Microscopy section, Laboratory Service Unit (LSU), Suranaree University of Technology, Nakhon Ratchasima Province 30000, Thailand

[4]Omics Science and Bioinformatics Center, Faculty of Science, Chulalongkorn University, Pathumwan, Bangkok 10330

[5]Center of Excellence in Biodiversity Research, Mahasarakham University, Maha Sarakham, 44150, Thailand.

* Corresponding author:
Onuma Kaewkla
Email address: onuma.k@msu.ac.th

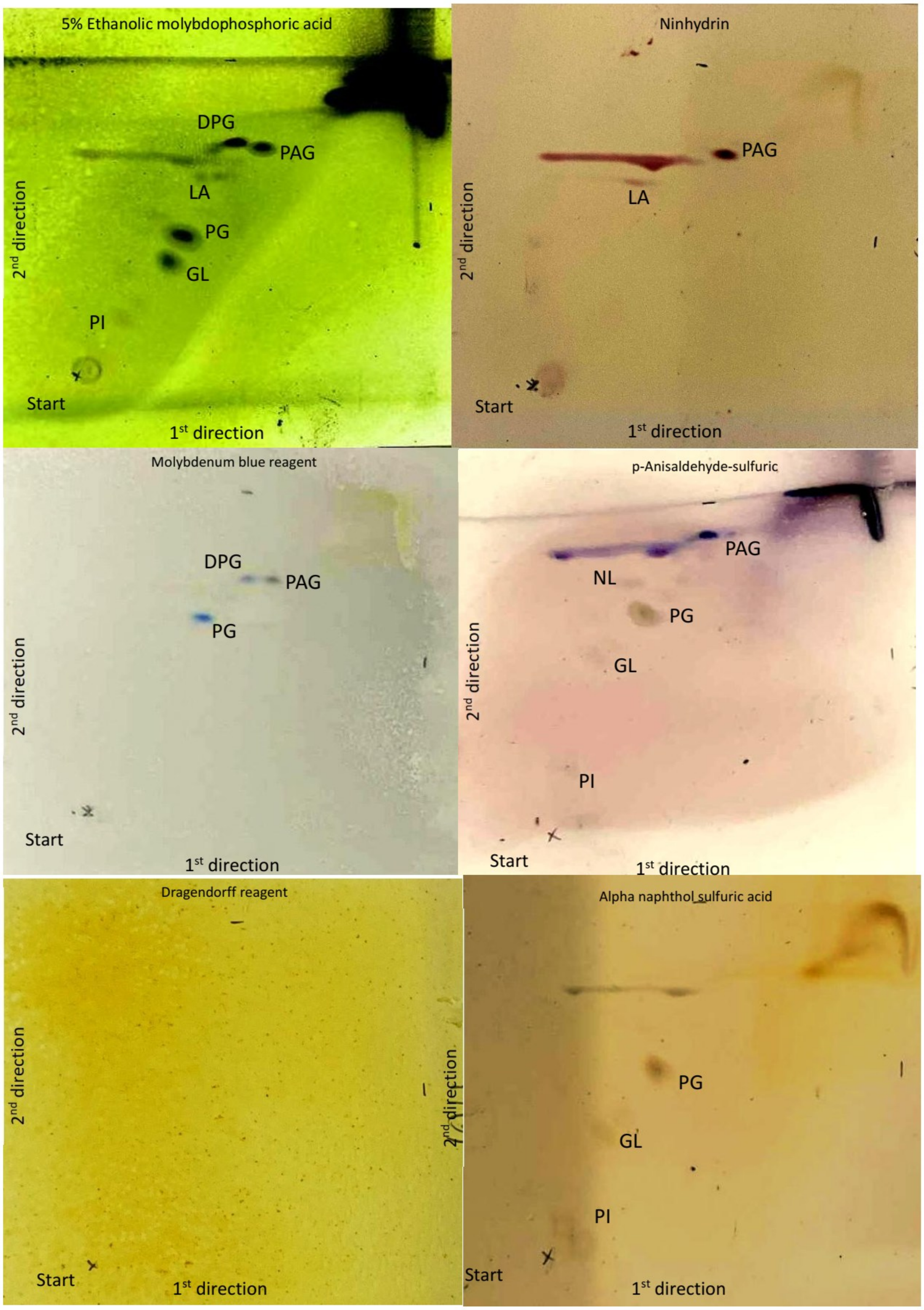
5% Ethanolic molybdophosphoric acid
DPG
PAG
LA
PG
GL
PI
Start
1st direction
2nd direction
Ninhydrin
PAG
LA
Start
1st direction
2nd direction
Molybdenum blue reagent
DPG
PAG
PG
Start
1st direction
2nd direction
p-Anisaldehyde-sulfuric
PAG
NL
PG
GL
PI
Start
1st direction
2nd direction
Dragendorff reagent
Start
1st direction
2nd direction
Alpha naphthol sulfuric acid
PG
GL
PI
Start
1st direction
2nd direction

A)

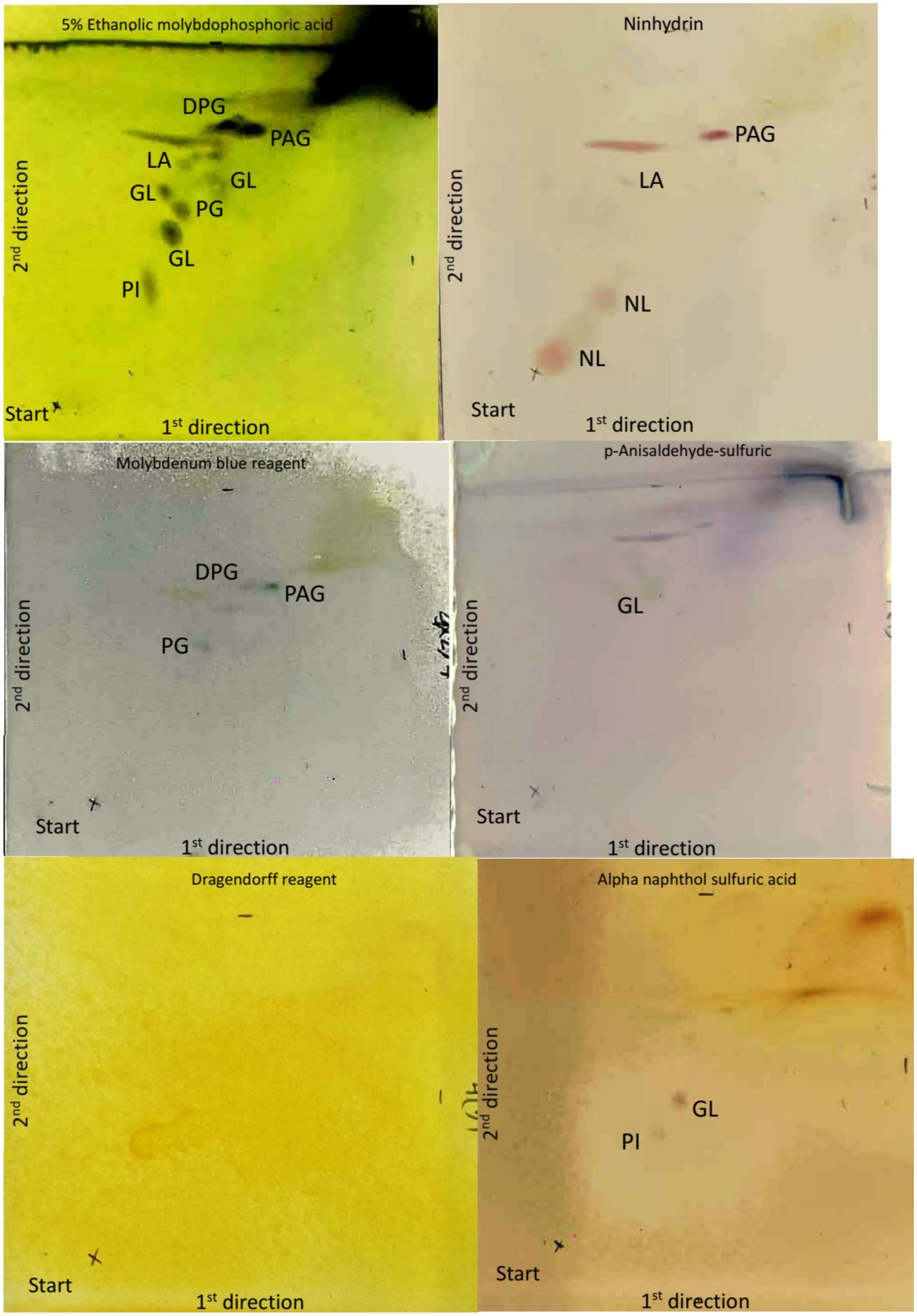
5% Ethanolic molybdophosphoric acid
DPG
PAG
LA
GL
GL
PG
GL
PI
2nd direction
Start
1st direction
Ninhydrin
PAG
LA
NL
NL
2nd direction
Start
1st direction
Molybdenum blue reagent
DPG
PAG
PG
2nd direction
Start
1st direction
p-Anisaldehyde-sulfuric
GL
2nd direction
Start
1st direction
Dragendorff reagent
2nd direction
Start
1st direction
Alpha naphthol sulfuric acid
GL
PI
2nd direction
Start
1st direction

B)

Figure S1 Two-dimensional thin-layer chromatography of polar lipids of

A) *Streptomyces kalasinensis* EKL1.1^{T} B) *Streptomyces phytorum* EKS8.28^{T}. Chloroform-methanol-water (65:25:4) was used in the first direction, followed by chloroform-acetic acid-methanol-water (40:7.5:6:2) in the second direction.

Abbreviations: DPG, diphosphatidylglycerol; PG, phosphatidylglycerol; PI, phosphatidylinositol; PAG, phospholipid with an amino group; GL, glycolipid; LA, lipid with an amino group; NL, not lipid.

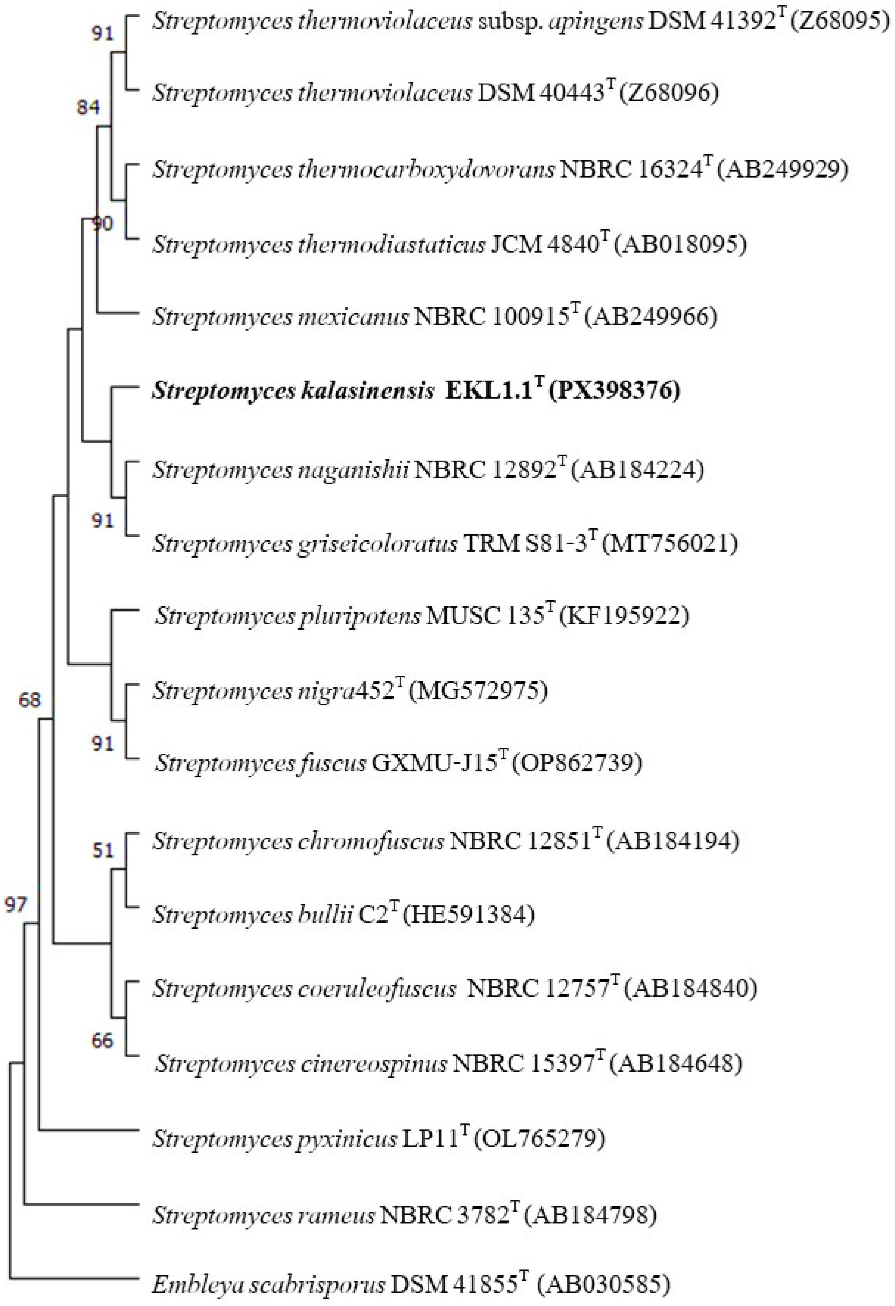

Figure S2 Neighbour joining phylogenetic tree based on 16S rRNA gene sequences (1360 bp) of *Streptomyces kalasinensis* EKL1.1^{T} and their closely related type strains in the genus *Streptomyces* and *Embleya scabrisporus* KM-4927^{T} as the outgroup. Bootstrap values based on 1000 replicates are shown at the branch nodes (the scale bar is not represented, as it is less than 0.000 changes per nucleotide).

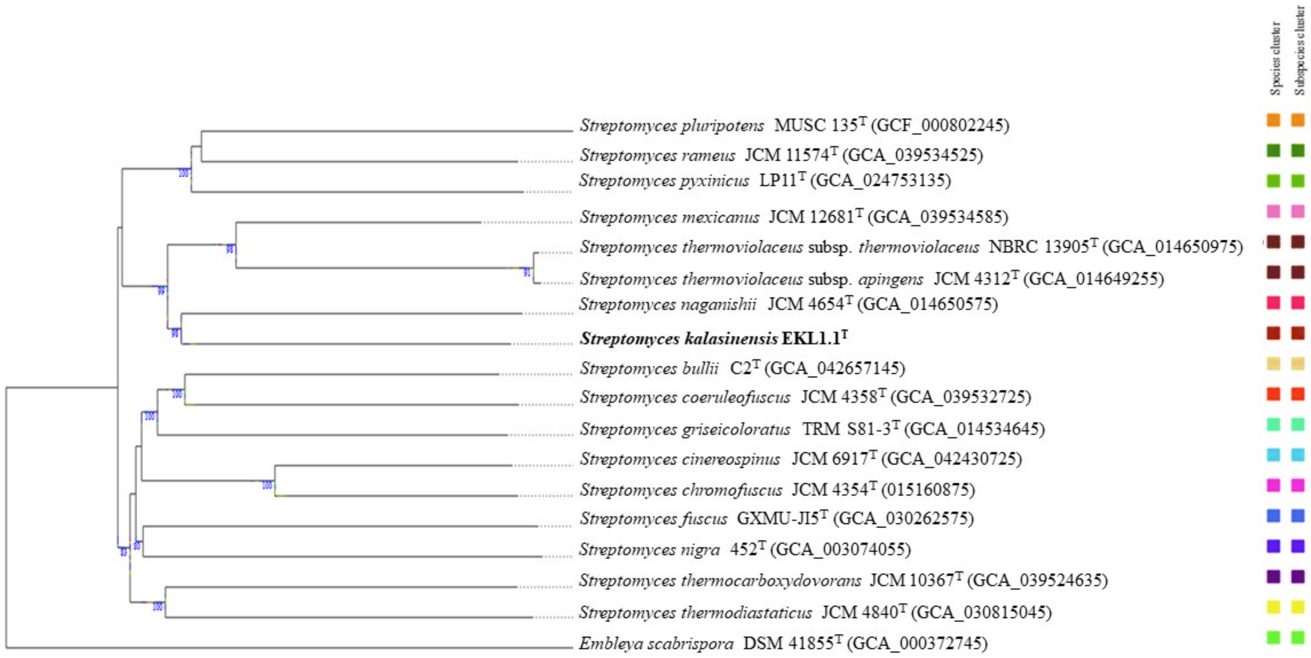

Figure S3 A phylogenomic tree based on a TYGS result showing the relationship between *Streptomyces kalasinensis* EKL1.1^T and their closely related type strains in the genus *Streptomyces* and *Embleya scabrisporus* KM-4927^T as the outgroup. The numbers above the branches are GBDP pseudo-bootstrap support values > 60% from 100 replications, with an average branch support of 81.3%. The tree was rooted at the midpoint (Farris, 1972). Leaf labels are annotated by affiliation to species and subspecies clusters (Meier-Kolthoff and Göker, 2019).

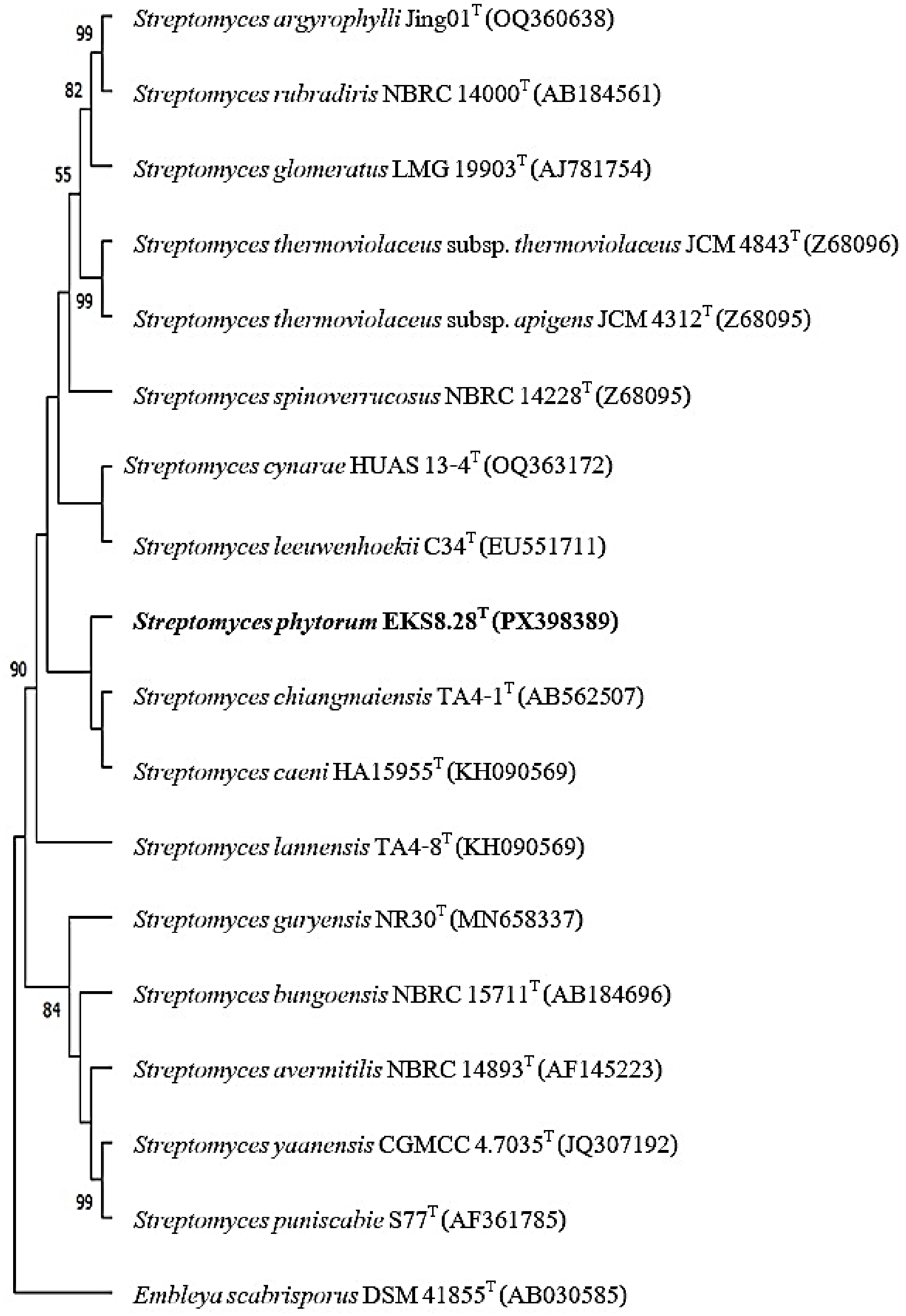

Figure S4 Neighbour joining phylogenetic tree based on 16S rRNA gene sequences (1356 bp) of *Streptomyces phytorum* EKS8.28^T and their closely related type strains in the genus *Streptomyces* and *Embleya scabrisporus* KM-4927^T as the outgroup. Bootstrap values based on 1000 replicates are shown at the branch nodes (the scale bar is not represented, as it is less than 0.000 changes per nucleotide).

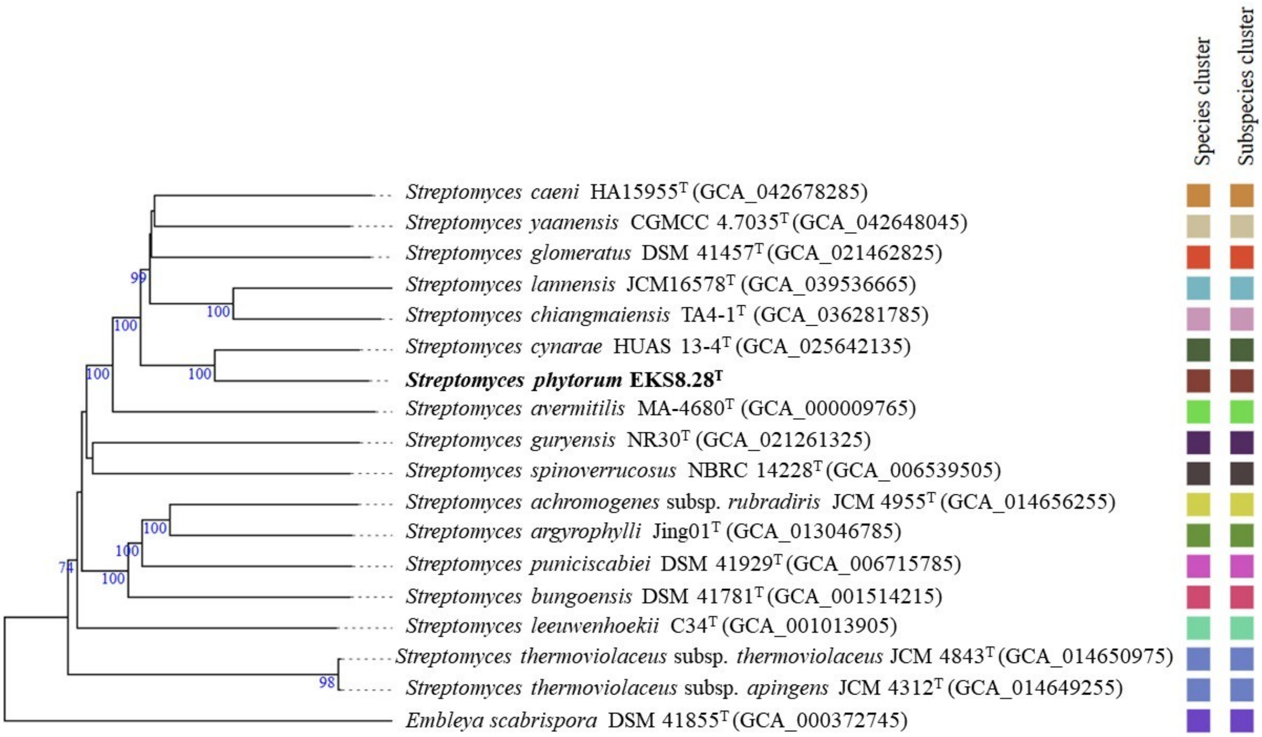

Figure S5 A phylogenomic tree based on a TYGS result showing the relationship between *Streptomyces phytorum* EKS8.28^{T} and their closely related type strains in the genus *Streptomyces* and *Embleya scabrisporus* KM-4927^{T} as the outgroup. The numbers above the branches are GBDP pseudo-bootstrap support values > 60% from 100 replications, with an average branch support of 85.6%. The tree was rooted at the midpoint (Farris, 1972). Leaf labels are annotated by affiliation to species and subspecies clusters (Meier-Kolthoff and Göker, 2019).

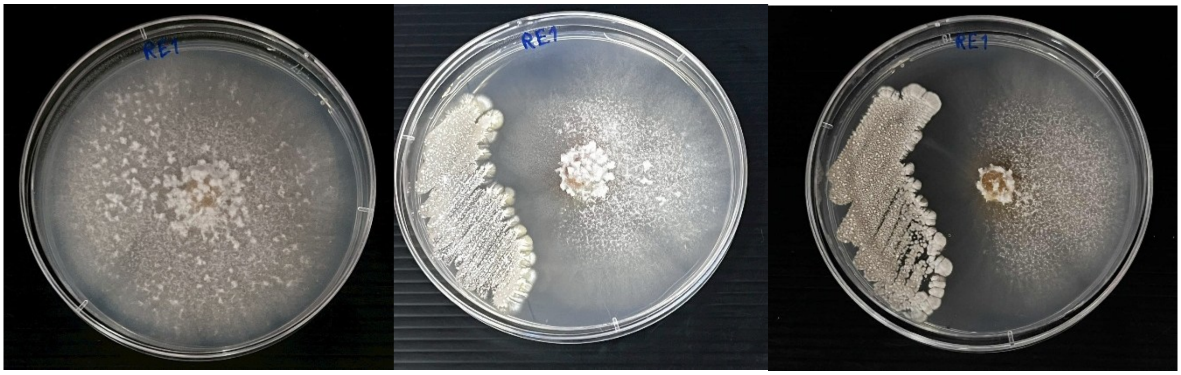

A B C

The dual culture test against *Fusarium* sp. RE1 A) Control plate without actinobacteria

B) Strain EKL1.1^T and C) Strain EKS8.28^T

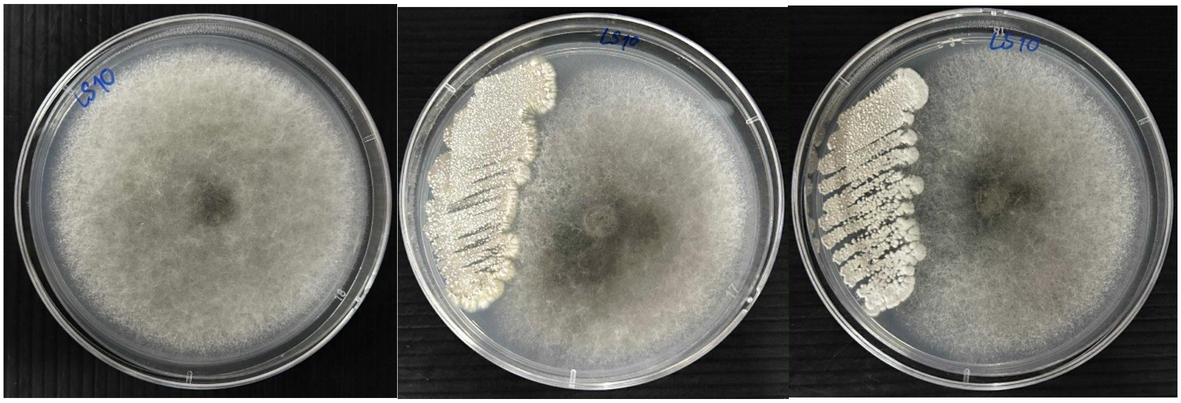

A B C

The dual culture test against *Colletotrichum* sp. LS10 A) Control plate without actinobacteria

B) Strain EKL1.1^T and C) Strain EKS8.28^T

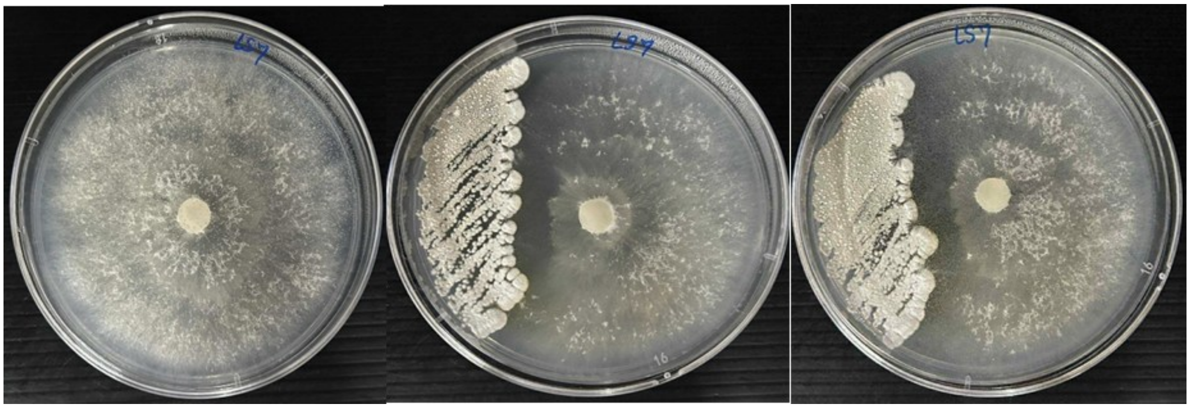

A B C

The dual culture test against *Diaporthe* sp. LS7 A) Control plate without actinobacteria B) Strain EKL1.1^{T} and C) Strain EKS8.28^{T}

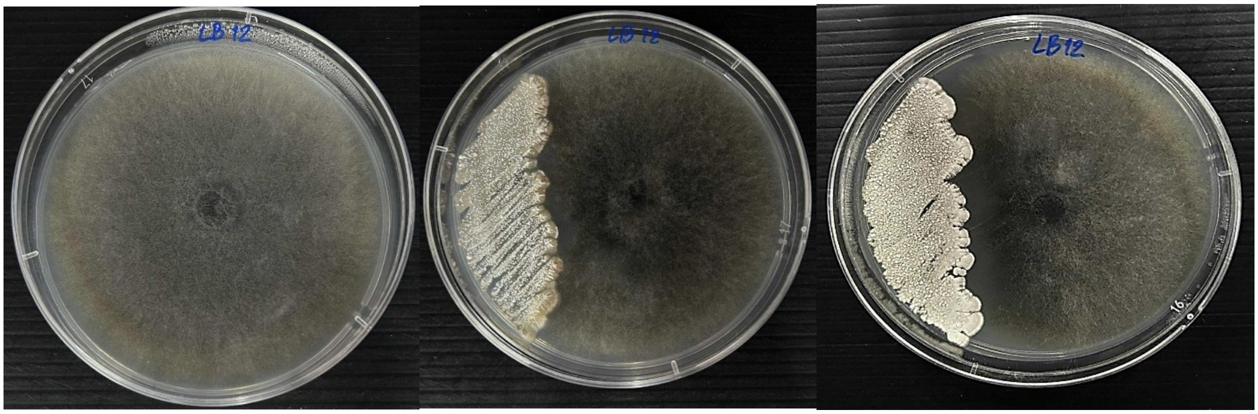

A B C

The dual culture test against *Curvularia* sp. LB12 A) control plate without actinobacteria B) Strain EKL1.1^{T} and C) Strain EKS8.28^{T}

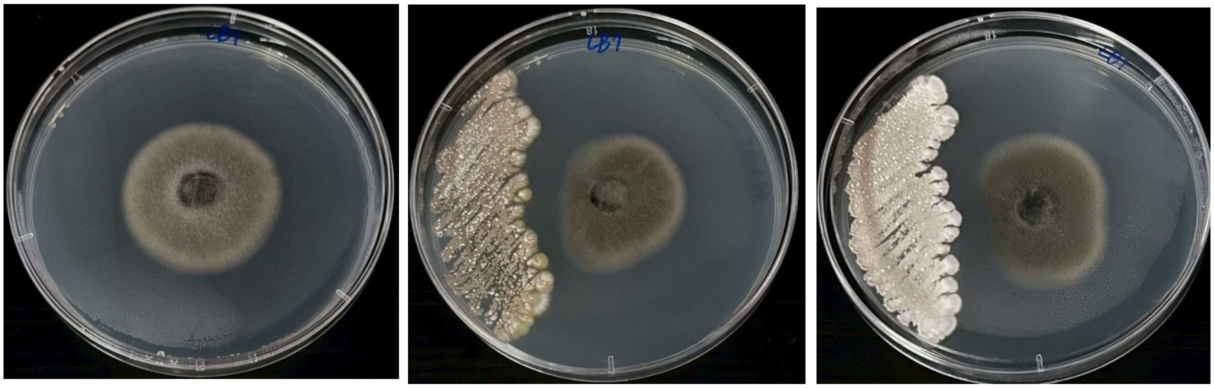

A B C

The dual culture test against *Cladosporium* sp. LB1 A) Control plate without actinobacteria B) Strain EKL1.1^{T} and C) Strain EKS8.28^{T}

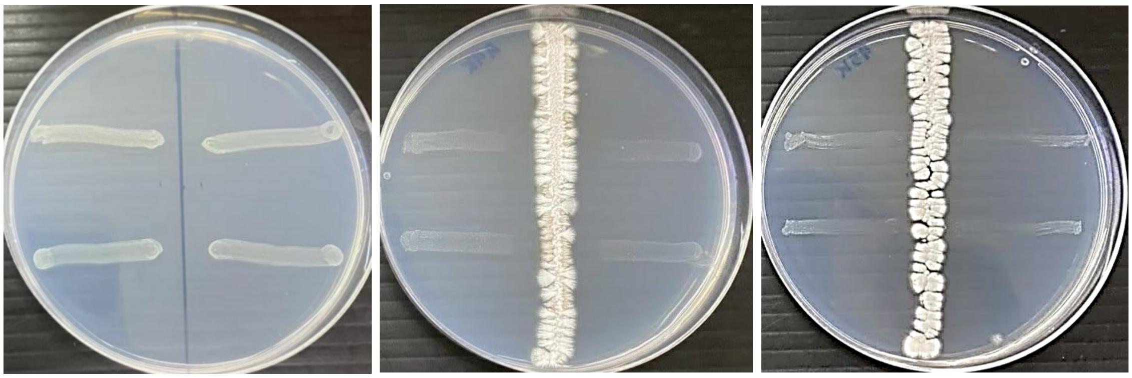

A B C

The dual culture test against *Ralstonia solanacearum* TISTR 2069 A) Control plate without actinobacteria B) Strain EKL1.1^{T} and C) Strain EKS8.28^{T}

Figure S6. The dual culture test of *Streptomyces* strains EKL1.1^{T} and EKS8.28^{T} against five fungal pathogens and one bacterial pathogen of eucalyptus.

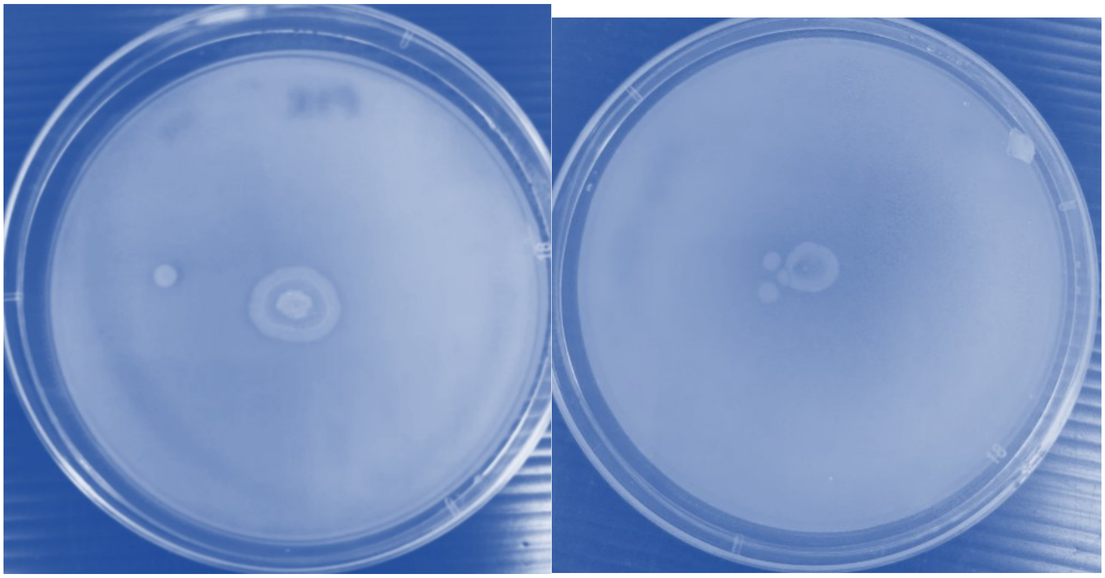

A B

Phosphate solubilization test A) strain EKL1.1^{T} and B) strain EKS8.28^{T} grown on the NBRIP medium for 10 days.

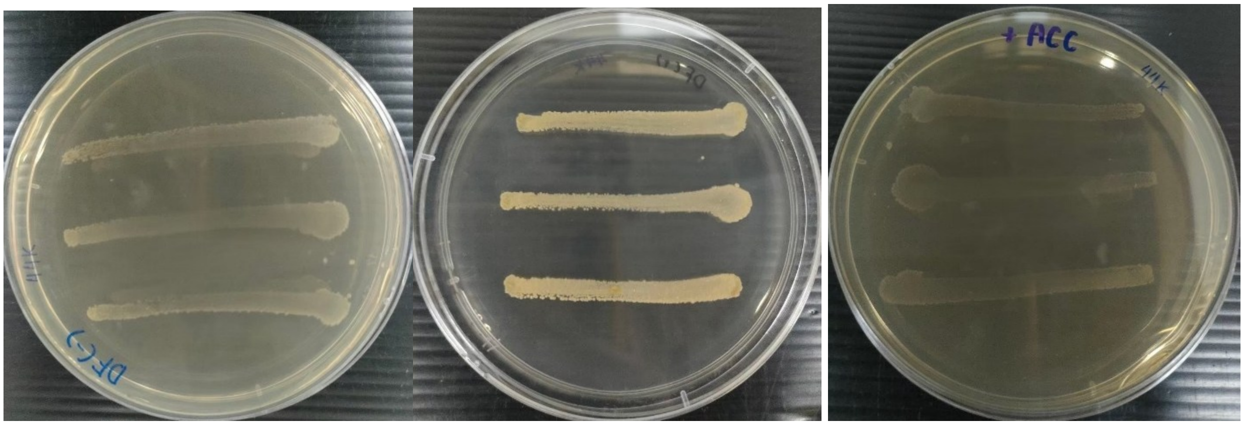

A B C

ACC deaminase test of strain EKL1.1^{T} grown on DF agar for 10 days A) DF agar without nitrogen source B) DF agar with nitrogen source C) DF agar with ACC

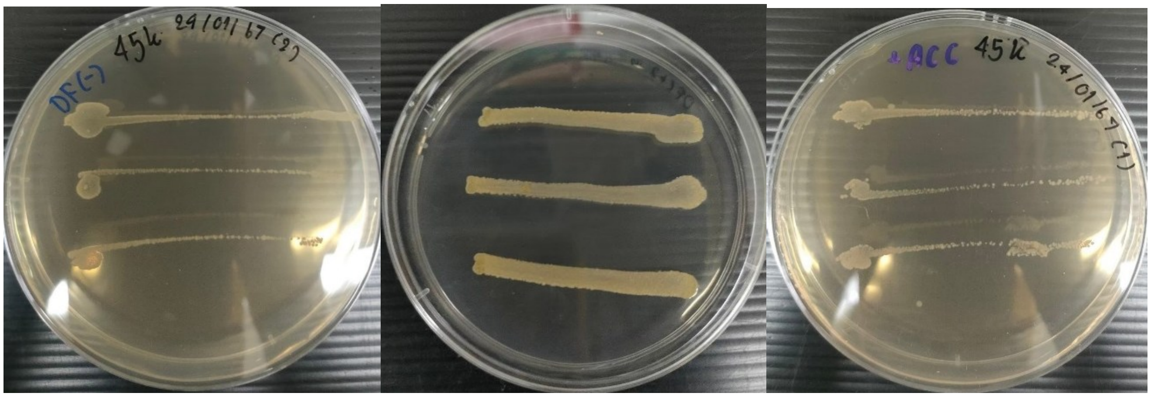

A B C

ACC deaminase test of strain EKS8.28^T grown on DF agar for 10 days A) DF agar without nitrogen source B) DF agar with nitrogen source C) DF agar with ACC

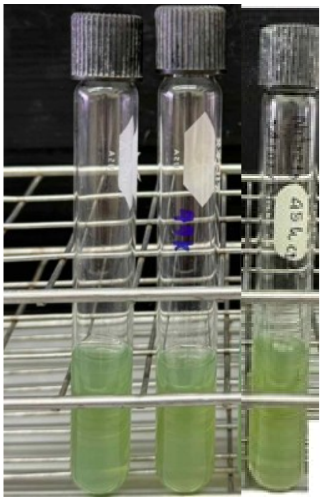

A B C

Nitrogen fixation test of A) control without actinobacteria B) strain EKL1.1^T C) strain EKS8.28^T grown on nitrogen-free semi-solid (NFb) agar for 10 days.

Figure S7 Plant growth promoting test of strains EKL1.1^T and EKS8.28^T

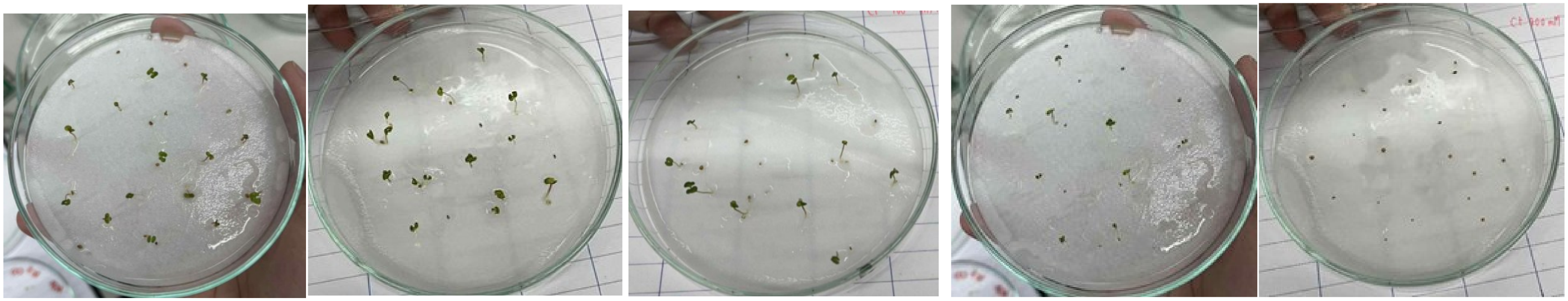

Control 0 mM NaCl 50 mM NaCl 100 mM NaCl 150 mM NaCl 200 mM NaCl

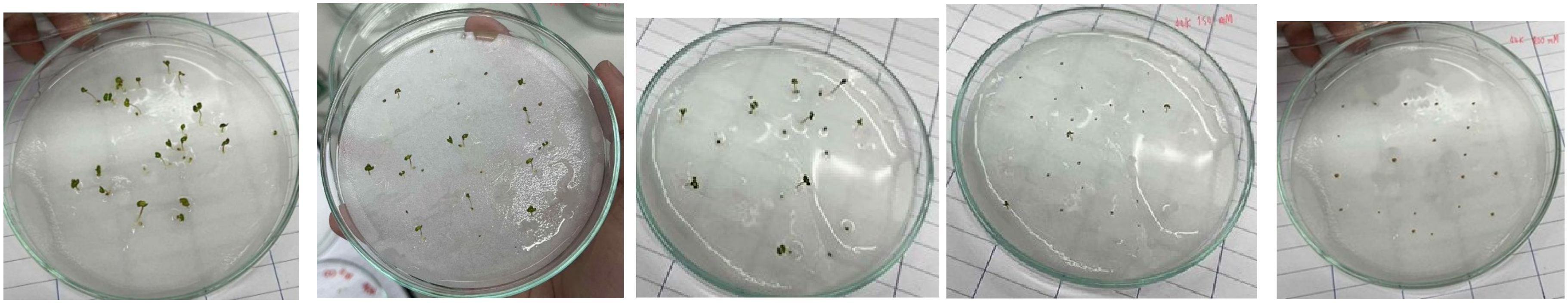

EKL1.1^{T} 0 mM NaCl 50 mM NaCl 100 mM NaCl 150 mM NaCl 200 mM NaCl

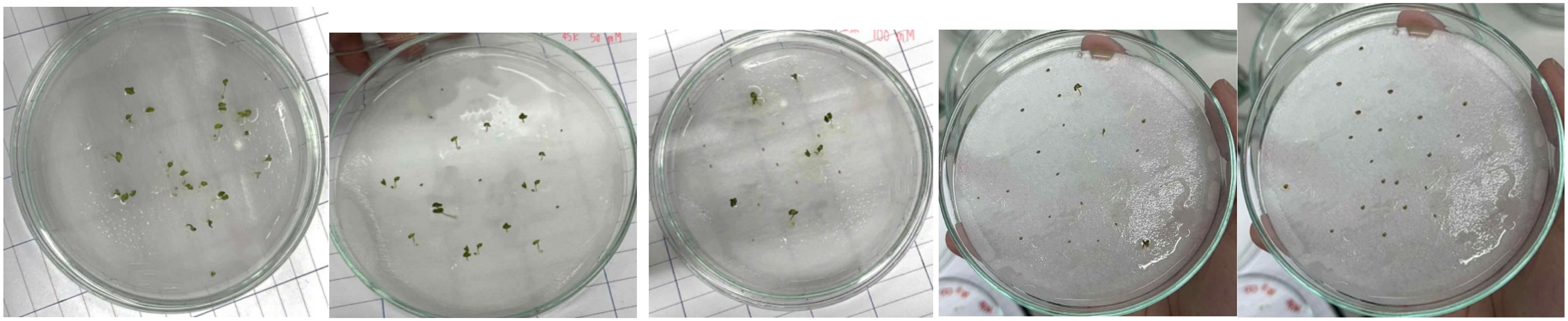

EKS8.28^{T} 0 mM NaCl 50 mM NaCl 100 mM NaCl 150 mM NaCl 200 mM NaCl

Figure S8 Seed germination test of eucalyptus seeds treated with control (water), strains EKL1.1^{T} and EKS8.28^{T} at 0, 50, 100, 150, and 200 mM NaCl and evaluated the result at day 8 of germination.

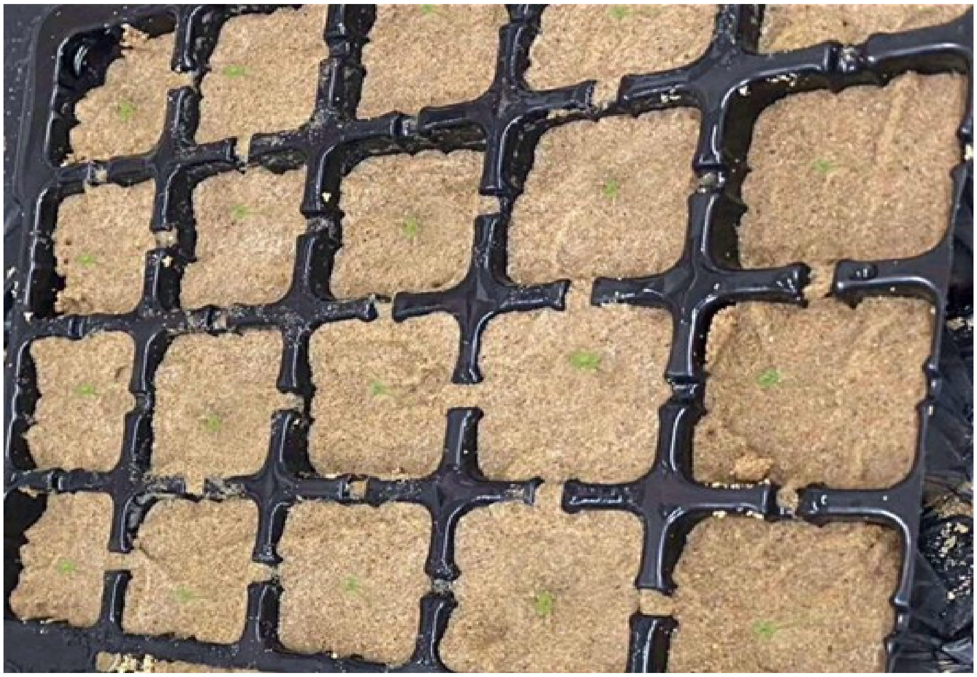

Control (water)

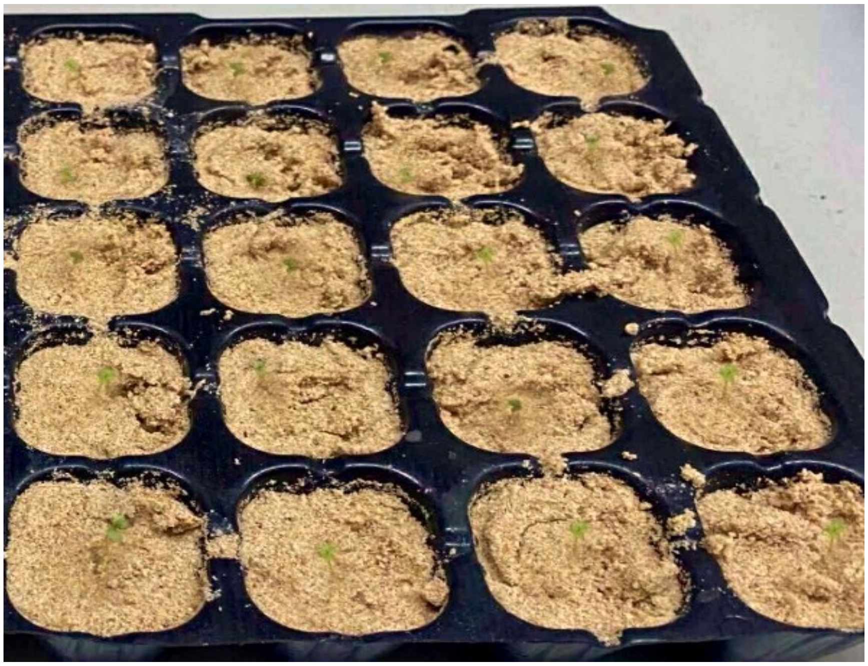

Strain EKL1.1^{T}

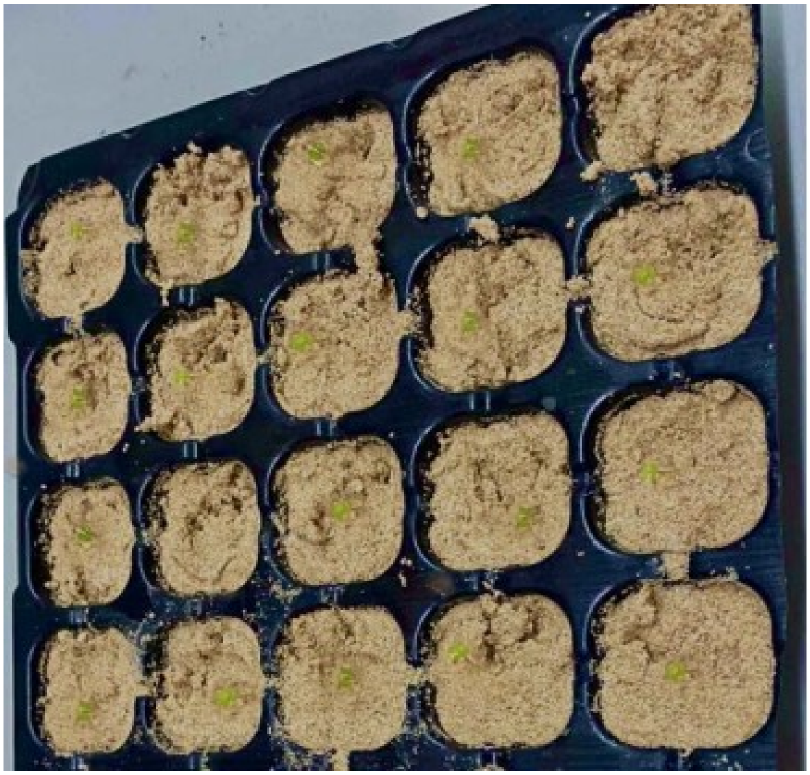

Strain EKS8.28^T

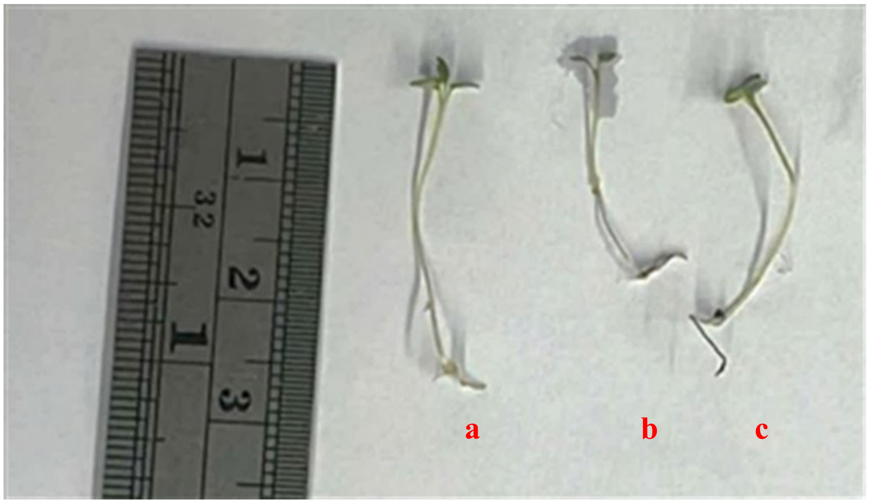

Figure S9 Plant growth promoting study of control (water), strains EKL1.1^T and EKS8.28^T to promote eucalyptus seedlings for 7 days after sowing a) Seedlings treated with strain EKL1.1^T

b) Seedlings treated with water c) Seedlings treated with strain EKS8.28^T at 14 days after sowing.
